# A high-toughness, rare-earth-free magnesium alloy with excellent biocompatibility profile: Integrated in vitro and in vivo characterization

**DOI:** 10.1016/j.mtbio.2026.103225

**Published:** 2026-05-13

**Authors:** Lukas Weber, Jelena Horky, Christopher Riedmüller, Carina Kampleitner, Eylem Acar, Mariangela Fedel, Claudia Höchsmann, Magdalena Eskinja, Stefan Mitsche, Bernhard Mingler, Laszlo Sajti, Gregor Mori, Manfred Bammer, Ingrid Walter, Stefan Tangl, Helga Bergmeister, Bruno K. Podesser, Marjan Enayati

**Affiliations:** a Center for Biomedical Research and Translational Surgery, Medical University of Vienna, Vienna, Austria; b Karl Donath Laboratory for Hard Tissue and Biomaterial Research, University Clinic of Dentistry, Medical University of Vienna, Vienna, Austria; c Ludwig Boltzmann Institute for Traumatology, The Research Center in Cooperation with AUVA, Vienna, Austria; d Austrian Cluster for Tissue Regeneration, Vienna, Austria; e RHP Technology GmbH, Wiener Neustadt, Austria; f Institute of Morphology, Department of Pathobiology, University of Veterinary Medicine Vienna, Vienna, Austria; g Chair of Technical and Analytical Chemistry, Montanuniversitaet Leoben, Leoben, Austria; h Institute of Electron Microscopy and Nanoanalysis (FELMI), Graz University of Technology, Graz, Austria; i Graz Centre for Electron Microscopy (ZFE), Graz, Austria; j Degree Program ‘High Tech Manufacturing’, Hochschule Campus Wien – University of Applied Sciences, Vienna, Austria

**Keywords:** Magnesium alloy, Biocompatibility, Inflammatory response, Biodegradability, Mechanical performance

## Abstract

Bioresorbable magnesium (Mg) alloys are promising alternatives to permanent implants in cardiovascular and orthopedic applications. Alloying with rare earth elements (REEs) can enhance corrosion resistance and mechanical strength, though their long-term biological behavior and biosafety are still being clarified. Herein, we compared the widely used REE-containing alloy WE43 with a previously self-designed REE-free alloy, ZX00 (Mg–0.6Zn–0.5Ca), focusing on biocompatibility, toxicity, degradation, and mechanical performance *in vitro* and *in vivo*. ZX00 demonstrated significantly higher fibroblast viability than WE43 *in vitro* (100.00% ± 9.30% vs. 43.00% ± 6.88%; p < 0.0001) and did not elicit inflammatory responses in primary human macrophages, while both alloys showed comparable hemocompatibility. After 5 months of subcutaneous implantation in rats, ZX00 and WE43 exhibited similar overall biocompatibility, with mild gas formation observed in X-ray imaging and histology. Although ZX00 degraded faster than WE43 according to micro-computed tomography and histological analyses, it retained markedly superior ductility after 5 months of implantation, due to uniform, non-localized corrosion. No systemic toxicity was detected for either alloy through histopathological assessment of multiple organs and hematological analyses. This study shows that the REE-free ZX00 alloy possesses the biological and mechanical requirements for demanding biomedical environments, positioning it as a promising candidate for cardiovascular applications and marking a first step toward a bioabsorbable material specifically designed for cardiac annuloplasty devices.

## Graphical abstract

**Figure:**
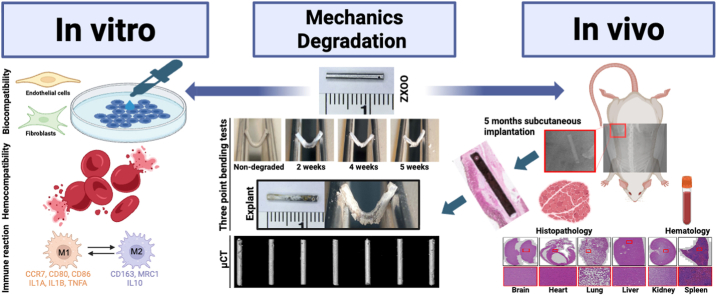

## 1 Introduction

Magnesium (Mg) alloys represent a promising bioabsorbable alternative to commonly used permanent implant materials for applications in which the implant is no longer needed after the healing or tissue remodeling phase, owing to their good biocompatibility, high specific strength, osteoconductivity, and strong resistance to oxidative stress [1]. However, they tend to corrode rapidly, which is accompanied by the formation of hydrogen gas and magnesium hydroxide (Mg(OH)_2_) (Equation (1)) as well as a concomitant increase in pH. Mg(OH)_2_ has low solubility and leads to the development of a protective layer. In simulated body fluid (SBF), cell culture media, or even living organisms, more complex processes occur, including the formation of a surface film of calcium phosphate and carbonates on the Mg alloy surface as well as the adhesion of cells and proteins [2]. Excessive corrosion of implants results in the premature loss of mechanical integrity, limiting their clinical applicability. In bone implantations, the evolution of hydrogen gas can also hinder osteoregeneration [3]. Similarly, in vascular stent applications, where the strut size is relatively small, controlling the corrosion rate of Mg to a desirable level remains a significant challenge [4].

$$Mg+2\;H_{2}O\;\to Mg{\left(OH\right)}_{2}+H_{2}$$

Equation (1): Mg degradation in contact with water.

To tailor both the corrosion rate and the mechanical properties of Mg, different approaches such as surface coatings and alloying have been applied. For surface coatings, various technologies, including chemical conversion coatings, plasma electrolytic oxidation, and laser surface modification, have been employed [5]. However, many coatings, such as biomimetic hydroxyapatite deposition, have not yet progressed to clinical studies in surgical applications [6,7]. Moreover, coatings can only slow the dissolution of an implant during the initial degradation phase.

Alloying approaches are generally categorized into rare earth element (REE)-containing and REE-free Mg alloys. Alloying can substantially improve the mechanical performance of Mg alloys, with each category offering distinct advantages and challenges. The most commonly used alloying elements include aluminum (Al), zinc (Zn), manganese (Mn), calcium (Ca), yttrium (Y), zirconium (Zr), and neodymium (Nd) [5]. The addition of REEs such as Y, Nd, cerium (Ce), gadolinium (Gd), and ytterbium (Yb) can substantially reduce the corrosion rate while providing greater mechanical strength than most REE-free Mg alloys. However, despite recent clinical trials supporting the safety of certain REEs at controlled concentrations, concerns regarding their biocompatibility and potential toxicity remain [8,9]. Some REEs may exhibit cytotoxic effects, therefore long-term tissue accumulation and possible impacts on organs such as the liver and kidneys must be evaluated across diverse patient populations [10,11]. One promising strategy moving forward is to combine alloying with coating approaches, particularly those based on REE-free alloys.

While in this work all materials were produced by using the classical approach of casting and extrusion, advanced techniques like for example laser powder bed fusion have demonstrated significant potential lately, especially when producing objects of complex geometry and smaller dimensions [12].

Recently, REE-free systems such as binary alloys (e.g., Mg–Zn or Mg–Ca) and ternary alloys (e.g., Mg–Zn–Ca) have been developed and combined with surface coating technologies [[13], [14], [15]]. These coatings employ various materials, including magnesium fluoride films, poly(lactic-co-glycolic acid) (PLGA), poly(carbonate urethane) urea (PCUU), and poly(ester urethane) urea (PEUU). In general, coating and surface modification help control ion release and improve both biocompatibility and degradation. Nevertheless, achieving consistent and reproducible surface coatings remains a major challenge, as poor coating quality can drastically reduce implant performance [16].

The Mg–Zn–Ca alloy system is regarded as one of the most promising candidates for bioresorbable implants, with broad applications in orthopedic surgery. Several studies have confirmed improved biocompatibility along with enhanced corrosion resistance when Mg is alloyed with Ca and/or Zn [17,18]. Moreover, these alloys have demonstrated excellent adhesion of bone-specific cells (such as MC3T3-E1 osteoblasts) and promising functional outcomes in large animal models [[19], [20], [21]].

In coronary stenting, Mg alloys have shown encouraging results in both preclinical and clinical studies. However, most major studies to date have been conducted with REE-containing Mg alloys, again raising concerns about potential toxicity during long-term use [9,22,23]. For example, the Magmaris system demonstrated that biodegradable Mg-REE implants can be broadly safe and effective but the specific clinical contribution of individual REEs remains uncertain. Current trials primarily assess major adverse events and offer limited insight into REE-specific biological effects. Consequently, dedicated and targeted studies are needed [8]. Although the Mg–Zn–Ca alloy system is primarily applied in orthopedic applications, our long-term research aims to adapt these alloys for cardiac valvular repair, specifically as annuloplasty ring. Apart from biocompatibility, a major aspect of these alloys is their mechanical characteristics, which depend on the clinical application. For instance, there is a fundamental difference between the use of materials (including Mg) as a vascular stent and for an annuloplasty ring. Mechanically, balloon-expandable stents experience radial forces exceeding 200 N during deployment, requiring unique mechanical properties, whereas forces acting on annuloplasty rings are substantially lower. In-plane forces on the mitral valve rarely exceed 5 N, as reported by Jedrzejczyk et al. [24]. This is well below the mechanical limits of our Mg samples, even after prolonged implantation.

Our study focuses on general biocompatibility, toxicological assessment, mechanical integrity, and controlled degradation behavior at this stage of investigation. One critical aspect of potential toxicity is the host immune response. Both innate and adaptive immunity play pivotal roles in the performance of Mg implants [25]. After implantation, acute inflammation recruits immune cells, followed by macrophage polarization (M1/M2) to direct healing, fibroblast activation, and neo-angiogenesis. Previously, our group developed a specific REE-free biodegradable ZX00 alloy (Mg–0.6 wt% Zn–0.5 wt% Ca) with outstanding mechanical strength through severe plastic deformation processing [[26], [27], [28]]. More recently, a ZX00 alloy of similar composition (Mg–0.45Zn–0.45Ca) has already been applied in a first-in-human study as a bone screw after extensive testing in a sheep model, demonstrating excellent biocompatibility, bone integration, and corrosion behavior [29]. Using the same Mg–Zn–Ca alloy in rat femoral implantations, Rahmati et al. demonstrated a strong effect on macrophage polarization toward the anti-inflammatory M2 subtype 10 days after implantation [30]. However, the cytocompatibility of the ZX00 alloy toward cardiovascular cells such as endothelial cells, together with its long-term immunomodulatory effects on immune cells like monocytes as well as its prolonged *in vivo* histopathological responses, remain insufficiently characterized.

In this study, we sought to systematically evaluate the biocompatibility, degradation behavior, and mechanically relevant performance of the REE-free biodegradable magnesium alloy ZX00 (Mg–0.6Zn–0.5Ca) and its variant containing a small addition of ytterbium (ZX00+, Mg–0.6Zn–0.5Ca–0.06 Yb). ZX00+ was specifically developed to reduce crack susceptibility during casting [31]. These materials were directly compared with the clinically established REE-containing alloy WE43 to assess whether REE-free Mg alloys can provide comparable biological compatibility and mechanical functionality while avoiding potential concerns associated with REE additions. This evaluation is intended as an initial step toward the long-term goal of using our ZX00 alloy for cardiac valvular repair applications.

## 2 Materials and methods

### 2.1 Mg alloy fabrication

The Mg alloys ZX00 (Mg–0.6Zn–0.5Ca, wt.%) and ZX00+ (Mg–0.6Zn–0.5Ca–0.06 Yb) were cast at Leichtmetallkompetenzzentrum Ranshofen GmbH, Austria, and their compositions were verified via spark emission spectroscopy. Ingots (50 mm diameter) were heat-treated and hot-extruded at 350 °C into rods with diameters of 12 or 6 mm. The same size of extruded rods of WE43 (Mg–4.1Y–3.7RE–0.5Zr; KG Fridman AB, Karlstad, Sweden) served as a reference. Disc samples (12 × 1 mm) from all alloys were prepared for *in vitro* biocompatibility testing, while rod samples (1.5 mm × 13 mm) of ZX00 and WE43 were used for *in vivo* studies and bending tests. For ZX00, rods were derived from 6-mm extrusions. A centered, 0.4-mm hole without tapering was laser-drilled at 1 mm from one end for implant fixation (KLUG Laserdienstleistungen, Hannover, Germany). All samples were ultrasonically cleaned in isopropanol and sterilized by gamma irradiation (25 kGy; Gammatron 1500, Mediscan GmbH & Co. KG, Seibersdorf, Austria).

### 2.2 Microstructural characterization

#### 2.2.1 Grain size measurements

Grain sizes of ZX00, ZX00+, and WE43 were determined according to ASTM E112 by Österreichisches Gießerei-Institut (Leoben, Austria). Discs of 1 mm thickness and 12 mm diameter were embedded, polished, etched using oxalic acid and analyzed by light microscopy.

#### 2.2.2 Electron backscatter diffraction (EBSD)

Disc-shaped samples (12 mm in diameter and 1 mm in thickness) were prepared by broad ion beam cross-sectioning in the central region along the planar disc face using a Gatan Precision Cross-Section System (Ilion+). During preparation, the samples were cooled to −150 °C to minimize microstructural alterations induced by ion-beam heating. On these cross sections EBSD investigations were performed using a Zeiss Ultra 55 field emission gun scanning electron microscope (FEG-SEM) equipped with a Thorlabs high-resolution scientific camera and OIM DC V7.3.1 acquisition software. An area of 300 μm × 300 μm was scanned with a step size of 0.4 μm. The microscope was operated at an accelerating voltage of 15 kV and a beam current of 7.2 nA. The acquired data were subsequently analyzed using OIM Analysis V9.1 software.

### 2.3 *In vitro* degradation experiments

To assess degradation, ZX00 and WE43 pins were incubated in SBF27 at 37 °C for 2, 4, and 5 weeks, as described previously [32]. The SBF, buffered with Tris-HCl (pH 7.40 ± 0.05), replicated the ionic composition of human plasma. Three to four pins were suspended in 250 mL of SBF per bottle using thin polymer filaments to prevent contact. Bottles were sealed with Parafilm (Bemis Company Inc., Neenah, WI, USA) to minimize evaporation and excessive pH drift. Because Mg degradation elevated the pH, the SBF was renewed weekly. At each timepoint, samples were removed, dried, and examined through light microscopy (Zeiss Axio Imager.M2m). Mass loss was quantified by weighing samples before and after incubation.

### 2.4 Mechanical testing

Three-point bending tests were performed on original and degraded ZX00 and WE43 pins to evaluate load-bearing capacity and toughness. Tests were conducted on a Shimadzu Servopulser EHF-UV050K1 universal testing machine using 3-mm diameter support rods, with a span of 7.4 mm between the two lower rods. Thereby we aimed to assess the practical applicability of the alloy by performing mechanical tests on partially degraded samples. The upper rod was displaced at 0.005 mm/s until sample fracture or, in highly ductile specimens, until geometric constraints required termination. Force and displacement were continuously recorded, and the maximum force, corresponding stroke, and end of the elastic regime (defined as the deviation from linearity indicating the onset of plastic deformation) were determined. The purpose of the measurements was not to determine basic material properties of the alloys by relating the actual diameters of the individual degraded samples to the measured force, but rather to investigate the loss of load-bearing capacity due to degradation which is linked to the overall loss of material as well as to the homogeneity of degradation. To investigate the toughness or overall robustness of the alloys after degradation, the areas below the force-displacement curves were determined by numerical integration. This is analogous to the definition of tensile toughness as the area under the stress–strain curve in a tensile test [33] but cannot be compared to standard fracture toughness tests.

Fracture surfaces were analyzed using light microscopy (Zeiss Axio Imager.M2m) and ImageJ software (v1.53k) [34]. For nonfractured samples, the diameter at the point of maximum bending was measured. In samples degraded for 2 weeks, end-surface areas were additionally assessed from polished cross-sections for comparison.

### 2.5 Electrochemical measurements

Electrochemical measurements were performed using a conventional three-electrode setup with the investigated alloys serving as working electrode, platinum as counter electrode and the saturated calomel electrode (SCE) as reference electrode. The setup was connected externally to the potentiostat ZRA R600 Gamry (Gamry, Warminster, USA) which was utilized for all tests. All tests were carried out at 37 °C in SBF solution. Open circuit potential (OCP) of each alloy was monitored continuously for 24 h prior to the potentiodynamic polarization scan to ensure that a stable value was reached. Potentiodynamic curves were obtained by polarizing the working electrode in the range from −500 mV to +500 mV respective to the OCP with the scan rate of 0.05 mV/s. Each test was repeated in triplicate to ensure reliability of the experimental data. Generated potentiodynamic curves were evaluated by Tafel extrapolation to determine the corresponding current density and corrosion rate.

### 2.6 Cytocompatibility assays

#### 2.6.1 Alloy extract preparation and characterization

Cytocompatibility was assessed using Mg alloy extracts prepared by immersing Mg discs in endothelial cell growth medium-2 (EGM-2; Lonza Group AG, Basel, Switzerland) for 72 h at 37 °C, in accordance with ISO 10993, with a surface area-to-volume ratio of 1.25 cm^2^/mL.

The chemical composition of extracts after 24 and 72 h of incubation was analyzed for major metallic elements (including REEs), selected semi-metals, and nonmetals. Analyses were performed through inductively coupled plasma mass spectrometry (ICP-MS; NexION 300D, PerkinElmer Inc., Waltham, MA, USA; Seibersdorf Labor GmbH, Seibersdorf, Austria) according to ISO 17294-2 and inductively coupled plasma optical emission spectrometry (ICP-OES; OPTIMA 7300 DV, PerkinElmer Inc.; Seibersdorf Labor GmbH, Seibersdorf, Austria) according to ISO 11885.

#### 2.6.2 *In vitro* biocompatibility: cell proliferation, live/dead assays, and scanning electron microscopy (SEM)

Cell viability was assessed using human umbilical vein endothelial cells (HUVECs; Lonza Group AG, Basel, Switzerland) and human foreskin fibroblasts (HFF-1; ATCC, Manassas, VA, USA). HUVECs were cultured in EGM-2 medium, and HFF-1 cells in Dulbecco's Modified Eagle Medium (Gibco, Thermo Fisher Scientific Inc., Waltham, MA, USA); both media were supplemented with 10% fetal bovine serum (Sigma-Aldrich, St. Louis, MO, USA) and 1% penicillin/streptomycin (Gibco, Thermo Fisher Scientific Inc.).

Cells were seeded in 24-well plates and incubated for 24 and 72 h with Mg alloy extracts at two concentrations: 50% (1:1 dilution with the respective culture medium) and 100% (undiluted extract). To further evaluate the influence of extract preparation, four conditions were tested, including pH adjustment and centrifugation, as summarized in Table 1.

**Table 1:** Mg extract conditions used for cytocompatibility tests.

Condition	Degradation byproducts	pH adjustment
1	Without	Yes
2	Without	No
3	With	Yes
4	With	No

Cell viability was quantified using XTT assay (2,3-bis(2-methoxy-4-nitro-5-sulfophenyl)-2H-tetrazolium-5-carboxanilide inner salt; Santa Cruz Biotechnology, Dallas, TX, USA) and measured with a Spark multimode microplate reader (Tecan Austria GmbH, Grödig, Austria). For live/dead staining, cells were incubated with Calcein AM (1 μg/mL; Biotium, Fremont, CA, USA) and propidium iodide (2.5 μg/mL; Sigma-Aldrich GmbH, Vienna, Austria) for 30 min, followed by three phosphate-buffered saline (PBS) washes and fixation with paraformaldehyde (Sigma-Aldrich GmbH, Vienna, Austria). Imaging was performed using an LSM700 confocal microscope (Carl Zeiss AG, Munich, Germany).

For SEM analysis, cells were fixed overnight at 4 °C in 2.5% glutaraldehyde (Sigma-Aldrich GmbH, Vienna, Austria), dehydrated through a graded ethanol series, and incubated overnight in hexamethyldisilazane (Sigma-Aldrich GmbH, Vienna, Austria). After chemical drying, samples were sputter-coated with a 10-nm gold layer and examined using a Zeiss EVO 10 scanning electron microscope (Carl Zeiss AG, Munich, Germany).

#### 2.6.3 Macrophage polarization and inflammatory response assessments

##### 2.6.3.1 Isolation and differentiation of monocytes

Peripheral blood mononuclear cells (PBMCs) were isolated from venous blood of healthy male and female donors (approved by the ethics committee: EK-NR.: 2321/2020), as previously described [35]. Following centrifugation with Ficoll Paque Plus (Cytiva, Marlborough, MA, USA), PBMCs were differentiated into macrophages using macrophage colony-stimulating factor (M-CSF; BioLegend Inc., San Diego, CA, USA) at a concentration of 50 ng/mL in RPMI 1640 (Sigma-Aldrich GmbH, Vienna, Austria) supplemented with 10% fetal bovine serum. The medium containing 50 ng/mL M-CSF was replaced after 3 days of culture, and cells were cultured for 5 days.

##### 2.6.3.2 Real-time quantitative polymerase chain reaction (qPCR)

Macrophages were cultured for 24 or 72 h in the presence of ZX00 and WE43 alloy extracts at two concentrations (50% and 100%). Total RNA was extracted using the RNeasy Mini Kit (Qiagen, Valencia, CA, USA). Complementary DNA was synthesized with the QuantiTect Reverse Transcription Kit (Qiagen, Valencia, CA, USA). Real-time qPCR was performed using a Roche LightCycler 480 (Roche AG, Basel, Switzerland). Primer sequences are provided in Supplementary Table 2. Furthermore, to assess macrophage polarization, the CCR7/CD163 gene expression ratio was calculated as an index of the M1/M2 response. CCR7 and CD163 were selected as representative markers of M1 and M2 macrophage phenotypes, respectively. Ratios greater than 1.0 indicate M1 predominance, whereas values below 1.0 indicate an M2-associated response. The ratio was calculated individually for each blood donor.

##### 2.6.3.3 Immunofluorescent stainings

Primary human monocytes were isolated and differentiated as described in Section 2.6.3.1. After 24 h of incubation with ZX00 extracts (50% and 100%), cells were washed with PBS, fixed in 4% paraformaldehyde, and permeabilized with 0.1% Triton X-100 (Sigma-Aldrich, St. Louis, MO, USA). Primary antibodies (Supplementary Table 3) were applied for 1 h, followed by 1 h of incubation with secondary antibodies. Stained cells were mounted on glass slides using a fluorescent mounting medium containing DAPI (1:1000; Sigma-Aldrich, St. Louis, MO, USA) and imaged with an LSM700 confocal microscope (Carl Zeiss AG, Munich, Germany). Furthermore, immunofluorescence images were quantified using QuPath software (version 0.6.0) [36]. Total cell numbers within each region of interest (ROI) were determined by nuclear counting. CD68 positive cells were identified as macrophages, and the number of CCR7 or CD163 positive cells was quantified within the CD68 positive population. Macrophage polarization was assessed by calculating the proportion of CCR7 or CD163 positive cells relative to the total number of CD68 positive cells (CCR7/CD68 and CD163/CD68 ratios), in each individual sample.

### 2.7 Hemocompatibility assays

#### 2.7.1 Blood clotting assay

Venous blood from healthy male (n = 2) and female (n = 2) donors was collected in citrate tubes (ethical approval: EK Nr. 2321/2020) and recalcified with 10 mM CaCl_2_ (Sigma-Aldrich GmbH, Vienna, Austria). Mg discs were weighed before incubation in recalcified blood at 37 °C for 1 h. After incubation, blood was removed, and discs with adherent clots were reweighed. Glass slides were used as positive controls.

#### 2.7.2 Hemolysis assay

Blood from seven healthy donors was collected as described in Section 2.6.3.1 and diluted 1:5 with 0.9% NaCl. Mg discs were incubated with diluted blood at 37 °C for 1 h, after which samples were removed and the blood was centrifuged (3000 rpm, 5 min). Plasma absorbance was measured at 541 nm using a Spark multimode microplate reader (Tecan Austria GmbH, Grödig, Austria). The hemolysis ratio was calculated according to Equation (2). Blood diluted 1:5 with distilled water served as the positive control, whereas blood diluted with 0.9% NaCl without Mg served as the negative control.

$$Hemolysis\;ratio\;\left(\%\right)=\frac{{Abs}_{s}-{Abs}_{neg}}{{Abs}_{pos}-{Abs}_{neg}}x100$$

Equation (2): Formula for calculating the hemolysis ratio. Abs_s_: Absorbance of sample; Abs_neg_: Absorbance of negative control; Abs_pos_: Absorbance of positive control.

### 2.8 Animal experiments

#### 2.8.1 Ethical considerations

All animal experiments were approved by the local animal ethics commission of the Medical University of Vienna and subsequently by the Austrian Federal Ministry of Education, Science and Research (License No. GZ:2022-0.121.461). All animals received humane care in accordance with the ARRIVE guidelines and EU Directive 2010/63/EU for animal experiments.

#### 2.8.2 Animals

Eight male Sprague–Dawley rats (9–11 weeks old; Charles River Laboratories Inc., Sulzfeld, Germany) were used. Animals were group-housed in a single P-type rabbit cage (Tecniplast S.p.A., Buggugiate, Italy) under a 12-h light/dark cycle with free access to standard rat chow ad libitum.

#### 2.8.3 Implant preparation and subcutaneous implantation

Sixteen Mg rods of WE43 and ZX00 were prepared as described in Section 2.1. For implantation, animals were anesthetized with 2%–3% isoflurane (Zoetis Österreich GmbH, Vienna, Austria) delivered via facemask. For additional analgesia, 30 mg/kg piritramide (Hameln Pharma GmbH, Hameln, Germany) was administered subcutaneously before surgery. Four rods of either WE43 or ZX00 were implanted subcutaneously on the back of each animal (n = 4 per alloy) and secured with a 6-0 Prolene suture (Johnson & Johnson Medical GmbH, Neuss, Germany). The skin was closed with a 4-0 Polysorb suture (Covidien GmbH, Neustadt, Germany).

#### 2.8.4 Radiological imaging (X-ray)

Radiographs were taken every 2 weeks under light isoflurane anesthesia using a digital X-ray device (Mobilett XP, Siemens Healthcare GmbH, Erlangen, Germany) to assess gas formation, degradation, and implant positioning.

#### 2.8.5 Explantation and tissue sampling

All animals were euthanized 5 months after implantation. Deep general anesthesia was induced with ketamine (350 mg/kg) and xylazine (40 mg/kg). Animals were placed in a supine position, and a thoracotomy was performed to expose the heart. Venous blood was collected from the right atrium, followed by exsanguination through the same site. Implants were excised together with the surrounding tissue.

#### 2.8.6 Histological and histomorphometric analyses

For histological analysis, specimens (ZX00, n = 7; WE43, n = 8) were fixed in 4% neutral buffered formalin, dehydrated in ascending ethanol concentrations, and embedded in light-curing resin (Technovit 7200 VLC + 1% benzoyl peroxide, Kulzer & Co., Wehrheim, Germany). Thin-ground longitudinal sections of approximately 100 μm were prepared through the center of each pin and stained with 2% toluidine blue and 0.3% pararosaniline according to the method developed by Karl Donath [37]. Both stained and unstained sections were scanned using an Olympus BX61VS digital microscopy system (DotSlide 2.4, Olympus, Tokyo, Japan) in two magnifications at resolutions of 0.64 and 0.32 μm per pixel.

Histomorphometric analysis was performed on unstained images using Adobe Photoshop (Adobe Inc., Mountain View, CA, USA) to evaluate pin degradation and hydrogen gas formation. For degradation assessment, the affected areas were measured and expressed as a percentage of the total implant area. Gas pockets surrounding the implants were quantified in the same manner. The thickness of the fibrous capsule was measured using QuPath open-source software (v0.6.0) [36].

#### 2.8.7 Mechanical testing of implanted Mg rods

The mechanical behavior of *in vivo* degraded rods was characterized via three-point bending tests as described in Section 2.4 (ZX00, n = 4; WE43, n = 5). Before testing, surrounding tissue was mechanically removed from the explanted Mg rods, which were then dried overnight.

#### 2.8.8 *Ex vivo* high-resolution micro-computed tomography (μCT)

ZX00 and WE43 samples (n = 7) were scanned using a SCANCO μCT 50 device (SCANCO Medical AG, Brüttisellen, Switzerland) at 55 kVp and 200 μA with a 0.5-mm Al filter. With a field of view of 35.2 mm, 850 projections per 180° were acquired at 250 ms integration time and reconstructed to an isotropic resolution of 20.7 μm. Scans were exported as DICOM stacks and processed in AMIRA software (Thermo Fisher Scientific, Waltham, MA, USA) for visualization and segmentation. Pin volume was semi-automatically segmented and expressed as a percentage of the original implant volume, determined by segmentation of pins before implantation.

#### 2.8.9 Immunohistochemical staining

Sections were dewaxed and rehydrated. Endogenous peroxidases were blocked with 0.6% hydrogen peroxide in methanol for 15 min and washed 10 times in distilled water. After antigen retrieval, nonspecific binding was blocked with either 1.5% normal goat serum (Dako, Glostrup, Denmark) or 1.5% normal rabbit serum (Dako, Glostrup, Denmark), depending on the source of the secondary antibody. Primary antibodies (Supplementary Table 4) were applied overnight at 4 °C, followed by incubation with secondary antibodies for 30 min at room temperature. Specific staining was visualized using 3,3′-diaminobenzidine in Tris-HCl buffer, pH 7.4 (Sigma-Aldrich, St. Louis, MO, USA). Sections were counterstained with Mayer's hematoxylin solution (Sigma-Aldrich, St. Louis, MO, USA), dehydrated in graded alcohols and xylene, and mounted with DPX (Sigma-Aldrich, St. Louis, MO, USA). Images were acquired with a TissueFAXS Plus slide scanner (TissueGnostics, Vienna, Austria).

#### 2.8.10 Real-time qPCR

Skin tissue surrounding the Mg rods was excised and homogenized using a TissueRuptor, followed by RNA extraction with the RNeasy Mini Kit (Qiagen, Valencia, CA, USA). Complementary DNA was synthesized with the QuantiTect Reverse Transcription Kit (Qiagen, Valencia, CA, USA). Real-time qPCR was performed using a Roche LightCycler 480 (Roche AG, Basel, Switzerland). The primer sequences are provided in Supplementary Table 2.

#### 2.8.11 Histopathological analysis

The heart, lungs, kidneys, spleen, liver, brain, and implant-surrounding skin were excised, fixed in 4% paraformaldehyde for 24 h, and then transferred to 70% ethanol. Organs were paraffin-embedded, sectioned, and stained with hematoxylin and eosin (H&E). Histopathological assessment was performed on collected tissue samples using a semi-quantitative grading system to evaluate the severity of observed findings. Each parameter was scored on a scale from 0 to 5, where 0 = no finding, 1 = minimal, 2 = slight, 3 = moderate, 4 = severe, and 5 = very severe. This grading system was applied to relevant pathological features, including inflammatory cell infiltration, tissue degeneration, necrosis, and fibrosis, where applicable. Only organs or tissue groups exhibiting detectable findings were included in the summarized results (Supplementary Table 5).

#### 2.8.12 Hematological analyses

Blood counts and blood chemistry, including a toxicological panel, were performed according to the standard operating procedures of IDEXX GmbH (Kornwestheim, Germany). Blood counts were conducted using an XN 1000V system (Sysmex Deutschland GmbH, Norderstedt, Germany), and all other analyses were performed with an AU480 system (Beckman Coulter Inc., Brea, CA, USA). In addition to histopathological analysis, systemic inflammatory status was assessed by calculating the neutrophil to lymphocyte ratio. This ratio was derived from hematological data by dividing the absolute neutrophil count by the absolute lymphocyte count for each sample. This parameter was used as an established indicator of systemic inflammatory response.

### 2.9 Statistical methods

Animals were considered the primary biological replicates (n = 4 per group). Multiple implants were placed within the same animal to maximize material and tissue level information in accordance with the 3Rs principle. Therefore, implants within a single animal were not treated as independent samples. In this case nested t-tests were performed to determine statistical significance. μCT and histological analyses included multiple implants per group (ZX00: n = 7, WE43: n = 8). Histological analysis followed the same grouping strategy and sample size, and implants within the same animal were not treated as independent samples.

All data are presented as mean ± standard deviation (SD). Statistical analyses were performed using GraphPad Prism version 10.2.0 (335) (GraphPad Software Inc., Boston, MA, USA). For comparisons between two groups, unpaired *t*-tests (two-tailed) or nested t-tests were conducted. For comparisons involving more than two groups, two-way analysis of variance with Šídák's multiple comparisons test was applied. Statistical significance was defined as *p* < 0.05.

## 3 Results

### 3.1 Microstructure and extract characterization

Grain size measurements and representative images of ZX00, ZX00+, and WE43 are presented in Supplementary Fig. 1. ZX00 exhibited an average grain size of 6.3 ± 1.2 μm, ZX00 + 4.7 ± 0.4 μm while WE43 had larger grains with 10.5 ± 1.1 μm average diameter. Furthermore, sporadic oxide particles were visible in the ZX alloys while the images of WE43 revealed numerous precipitates due to the low solubility of the REEs in the Mg matrix. More detailed microstructural analysis of ZX00 can also be found in a previous publication by Horky et al. [28].

EBSD revealed that the microstructure of WE43 was fully recrystallized and exhibited a homogeneous grain size distribution (Supplementary Fig. 2). In contrast, ZX00 displayed a heterogeneous microstructure composed of both deformed and recrystallized grains, with the recrystallized grains being significantly smaller than the deformed ones confirming the grain size measurements done by light microscopy. Furthermore, the crystallographic textures of the two alloys differed markedly. This was evidenced by the maximum intensity of the inverse pole figures derived from the EBSD data (Supplementary Fig. 3). ZX00 exhibited a strong texture whereas WE43 showed a comparatively weak texture.

The results of elemental screening of ZX00 and WE43 extracts using ICP-MS and ICP-OES are summarized in Table 2. Compared with the control medium (EGM-2), all extracts showed markedly elevated Mg concentrations. For ZX00, the alloying elements Zn and Ca were also significantly higher than those in controls. However, Mg and Ca concentrations decreased at later time points, likely due to precipitation of Mg- and Ca-containing byproducts.

**Table 2:** Chemical analyses of Mg alloy extracts after 24 or 72 h of incubation in EGM-2 medium. All results are expressed in mg/L.

	Mg	Zn	Ca	Na	K	P	Fe	Mn	Cu
**EGM-2 (control)**	240	<0.5	64	2960	180	29	0.28	<0.025	<0.025
**ZX00 24 h**	960	4.3	25	2770	180	8.1	0.25	<0.025	<0.025
**ZX00 72 h**	820	6.2	22	2780	180	4.2	0.24	<0.025	<0.025
**WE43 24 h**	1400	1.0	22	2750	180	5.2	0.28	0.044	0.048
**WE43 72 h**	1800	1.1	17	2820	180	4.5	0.25	0.041	0.039

Other medium constituents, including Na, K, and Fe, were unaffected by either alloy. Conversely, P concentrations were strongly reduced in all extracts. Trace amounts of Mn and Cu detected in WE43 extracts originated from minor alloy impurities, consistent with the material certificate.

### 3.2 Corrosion behavior

The recorded change of the open circuit potential of the alloys WE43 and ZX00 during the 24-h immersion period can be seen in Supplementary Fig. 4. After immersion the potential for both alloys increases rapidly from approximately – 1.90 V_SCE_ up to −1.50 V_SCE_ in the first 5 ks of measurement. The swift increase of potential to more positive values indicated the formation of a layer of corrosion products in contact with the SBF solution. Initially, the potential of WE43 appears to be slightly more negative compared to ZX00, suggesting a higher activity of the alloy. Nevertheless, after approximately 40 ks, the potentials of the alloys stabilize and reach identical values.

The potentiodynamic polarization curves of both alloys measured after stabilization period of 24 h are shown in Supplementary Fig. 5. The data estimated from the linear Tafel extrapolation of the measured curves including corrosion potential (E_corr_), corrosion current density (i_corr_) and cathodic Tafel slopes are listed in Supplementary Table 1. The determined i_corr_ value of the alloy ZX00 is slightly lower compared to the WE43 alloy while the E_corr_ was shifted for both alloys to the more noble values compared to the OCP. No passive zone was obtained for the two alloys. On the other hand, the cathodic slope of WE43 displays fluctuations in the current response.

### 3.3 Cytocompatibility

**Fig. 1:**
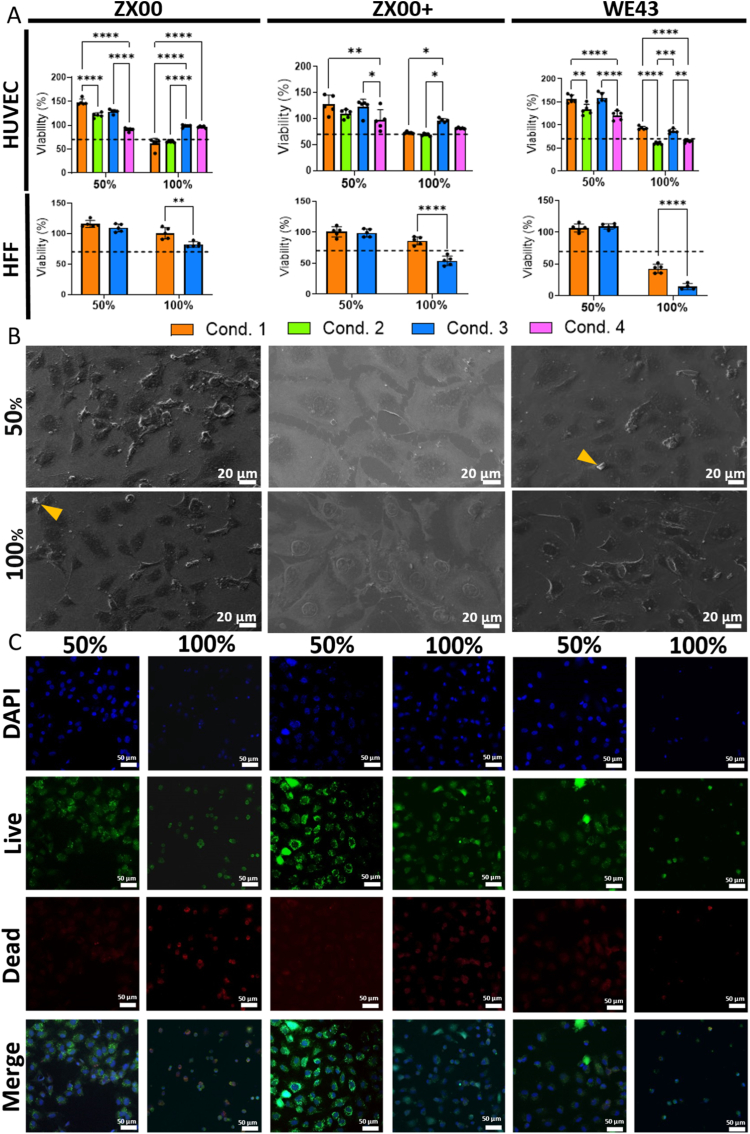
XTT cell viability, SEM, and live/dead staining. (A) XTT assays of three Mg alloys (WE43, ZX00, and ZX00+) using HUVECs and HFFs. Extracts were tested at 50% and 100% concentrations. For HUVECs, extracts were prepared under four conditions (Cond. 1–4) and incubated for 24 h (n = 5 per condition). For HFFs, only conditions 1 and 3 were used. The cytocompatibility threshold (70%) is indicated by a dashed line. (B) SEM micrographs (Cond. 3) of HUVECs incubated with ZX00, ZX00+ and WE43 extracts. Yellow arrows highlight Mg byproducts. (C) Live/dead staining of HUVECs incubated for 24 h with WE43, ZX00, or ZX00+ extracts (Cond. 3). Data are shown as individual points with mean ± SD. ∗*p* < 0.05, ∗∗*p* < 0.01, ∗∗∗*p* < 0.001, ∗∗∗∗*p* < 0.0001. (For interpretation of the references to colour in this figure legend, the reader is referred to the Web version of this article.)

All Mg alloys tested at 50% dilution showed cell viabilities above 70% (ranging from 76% to 173%) and were therefore classified as biocompatible according to ISO 10993 (Fig. 1A). In contrast, undiluted extracts (particularly those without pH adjustment) failed to meet the cytocompatibility threshold in HUVECs, underscoring the importance of pH regulation in experimental setups.

Consequently, pH-adjusted extracts were used for XTT assays with HFFs. Consistent with the findings in HUVECs, fibroblasts exposed to 50% extracts exhibited high viability (88%–126%). Under undiluted conditions, viability declined, and in the case of WE43, fell below the cytocompatibility threshold. The ZX00+ alloy (Mg–0.6Zn–0.5Ca–0.06 Yb) also showed reduced biocompatibility compared with ZX00, particularly in undiluted extracts. Due to this lower cell viability ZX00+ was not included in subsequent experiments.

SEM analysis revealed more viable cells in 50% than in 100% extracts, with visible extract byproducts (Fig. 1B). These results were corroborated by live/dead staining (Fig. 1C), which showed the highest Calcein AM positivity in 50% extracts, whereas 100% extracts consistently reduced cell viability.

### 3.4 Macrophage polarization and hemocompatibility

**Fig. 2:**
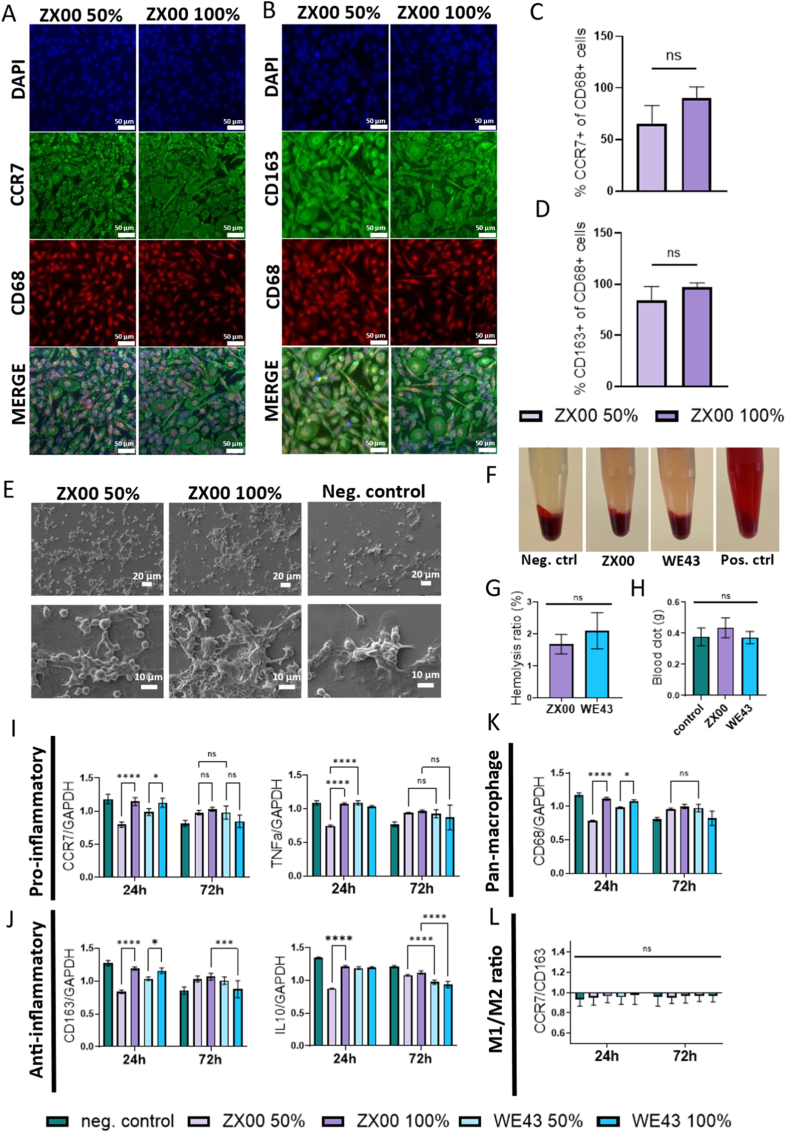
Macrophage polarization and hemocompatibility assays. (A) Immunofluorescence staining of macrophages with CCR7 as a proinflammatory marker and (B) CD163 as an anti-inflammatory marker. CD68 was used as a pan-macrophage marker. (C&D) Quantification of immunofluorescence images via presenting the percentage of CCR7 or CD163 positive cells within the CD68 positive macrophage population. (E) SEM micrographs of macrophages incubated for 24 h with ZX00 extracts or negative control (no Mg). (F) Representative images of blood after centrifugation following incubation with negative control (no Mg), ZX00, WE43, or positive control (distilled water). (G) Hemolysis assay after 1 h of incubation of blood with Mg alloys. (H) Blood clotting assay after 1 h of incubation of blood with Mg alloys (ZX00 and WE43) or control (no Mg). (I) Proinflammatory (M1) and (J) anti-inflammatory (M2) gene expression after 24 and 72 h of incubation with ZX00 and WE43 extracts (50% or 100%). (K) Gene expression analysis of CD68 expression. (L) Ratio of CCR7/CD163 gene expression. Data are presented as mean ± SD. ns = not significant, ∗*p* < 0.05, ∗∗*p* < 0.01, ∗∗∗*p* < 0.001, ∗∗∗∗*p* < 0.0001.

Immunofluorescence stainings revealed no significant pro- or anti-inflammatory responses. Comparison of the CCR7 proinflammatory marker (Fig. 2A) with the CD163 anti-inflammatory marker (Fig. 2B) showed no significant polarization toward either the proinflammatory M1 or anti-inflammatory M2 phenotype following incubation with ZX00 extracts. CD68 was used as a pan-macrophage marker. Macrophage polarization was assessed by calculating the proportion of CCR7-or CD163-positive cells relative to the total number of CD68-positive cells in each individual sample.

Quantification of the immunofluorescence stainings (Fig. 2C and D) revealed a slight increase in the CCR7/CD68 and CD163/CD68 proportions in the 100% extract condition. However, this effect was not statistically significant. Overall, no extract concentration dependent effects were observed, indicating the absence of a significant pro- or anti-inflammatory response. SEM analysis confirmed the morphological integrity of cells exposed to ZX00 extracts (Fig. 2E). As expected, macrophage polarization influenced cell shape: M2 macrophages exhibited elongated morphology, whereas M1 macrophages appeared round or flattened as described previously [38]. SEM micrographs displayed a mixture of both morphologies, consistent with the IF findings. qPCR analysis of proinflammatory (CCR7 and TNFα) and anti-inflammatory (CD163 and IL-10) markers was performed after 24 and 72 h of incubation with 50% and 100% extracts of WE43 and ZX00 (Fig. 2I–L).

Pan-macrophage CD68 expression showed no significant differences between WE43 and ZX00 with 100% extracts at either time point, indicating comparable and overall mild macrophage activation.

For pro-inflammatory markers (Fig. 2I), 100% extracts induced higher CCR7 and TNFα expression at 24 h in both alloys (except TNFα expression in WE43); however, this response was no longer significant at 72 h. Consequently, no significant differences in pro-inflammatory gene expression were observed between the alloys at the later time point, confirming the absence of a sustained pro-inflammatory response. Anti-inflammatory responses (Fig. 2J) were largely independent of extract concentration, except at 24 h when CD163 and IL-10 expression was significantly increased. Notably, after 72 h of incubation with 100% extract, ZX00 induced significantly higher CD163 and IL-10 expression than WE43, indicating a more pronounced anti-inflammatory response. Macrophage polarization assessed by the CCR7/CD163 ratio (Fig. 2L) revealed no significant differences between groups, with all conditions exhibiting a mild M2-like phenotype. Together, these findings confirm the absence of strong inflammatory activation of macrophages in response to ZX00 extracts.

Blood clotting assays revealed no significant differences in blood clot formation among ZX00, WE43, and controls (Fig. 2H), providing a preliminary indication of the material's compatibility with blood. In a photometric assay, hemolysis ratios for both alloys remained below 2.5% (Fig. 2F–G), confirming the absence of hemolytic effects.

### 3.5 *In vitro* degradation and mechanical testing

**Fig. 3:**
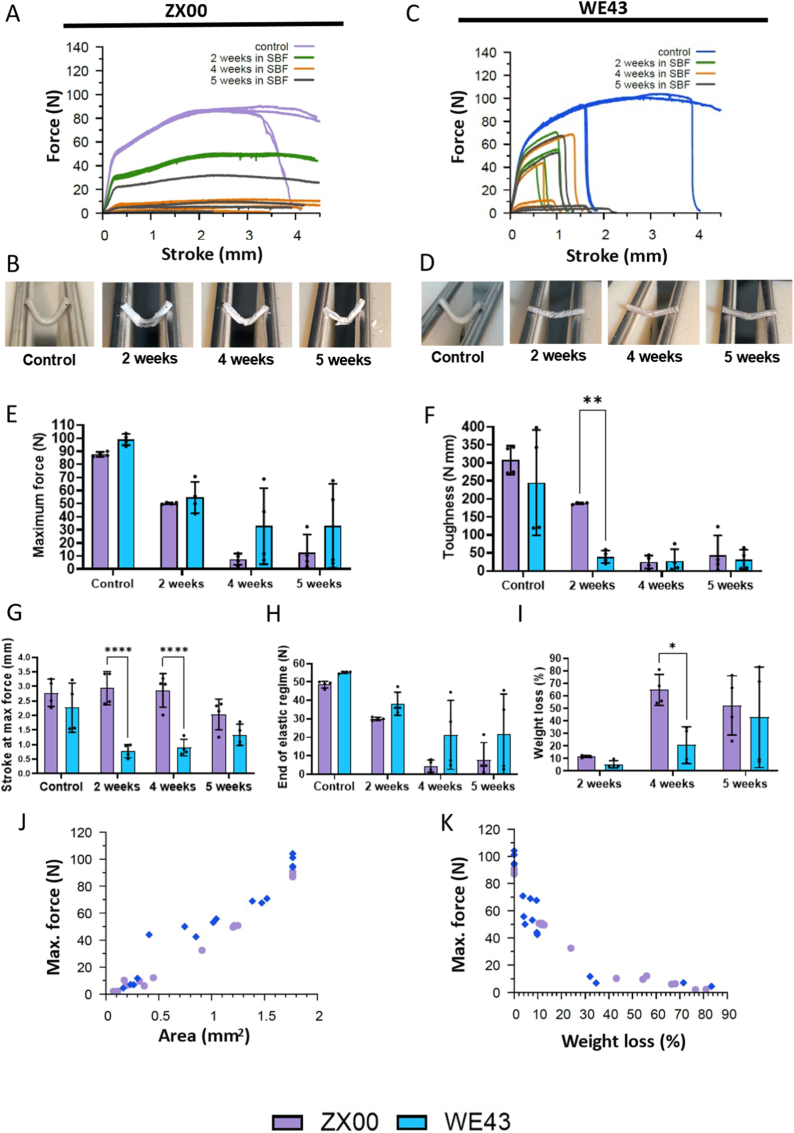
Mechanical testing of *in vitro* degraded samples. (A, C) Force–stroke curves from three-point bending tests of ZX00 and WE43 pins before and after degradation in SBF for different time periods. (B, D) Representative images of pins immediately after bending tests (E-H) Quantitative analysis of bending curves: (E) maximum force, (F) toughness (i.e., integrated area underneath the force-stroke curve), (G) stroke. at maximum force, and (H) end of the elastic regime (onset of plastic deformation). (I) Weight loss during *in vitro* degradation over time. (J) Correlation between maximum force and fracture surface area/cross-section area in the bent region. (K) Correlation between maximum force and weight loss. Data are presented as individual values with mean ± SD. ∗*p* < 0.05, ∗∗p < 0.01, ∗∗∗∗*p* < 0.0001.

Force–stroke curves (Fig. 3A–C), representative images (Fig. 3B–D), and mechanical characteristic graphs (Fig. 3E–H) showed that non-degraded control pins of both alloys (before SBF incubation) were highly ductile, with most specimens not fracturing within the maximum deflection of 4.5 mm. WE43 controls exhibited a higher maximum force and a slightly larger elastic regime than ZX00 controls. After degradation in SBF, mechanical performance was markedly altered. The most significant differences between the two alloys concerned the toughness, which was significantly higher in case of ZX00 after 2 weeks of degradation compared to WE43, as well as the ductility.

ZX00 retained high ductility and most samples did not fracture until the end of the test. Also, no reduction in stroke at maximum force relative to controls was observed. WE43, however, lost most of its deformability, and samples fractured at low deflection values (0.5–2.0 mm; Fig. 3G).

For both alloys, maximum force and the end of the elastic regime decreased following degradation. The maximum force correlated with fracture surface area or bent cross-sectional area (Fig. 3J), confirming that the reduced load-bearing capacity resulted from material loss and decreased pin diameter.

Weight loss was found to be lower in WE43 than ZX00 (Fig. 3I). However, after 5 weeks of degradation, the significance of these results was limited, as degradation products remaining on the pin surfaces could lead to underestimation of weight loss. Weight loss of individual samples was correlated with load-bearing capacity, i.e., the maximum force during the bending test (Fig. 3K). As expected, both alloys showed a rapid decline in maximum force as weight loss increased. Finally, the impact of sterilization on mechanical properties of the Mg alloys was examined, as such treatments could potentially alter material performance. Isopropanol cleaning followed by gamma irradiation did not significantly affect the mechanical properties of either alloy (Supplementary Fig. 6).

### 3.6 Small animal subcutaneous implantation study

**Fig. 4:**
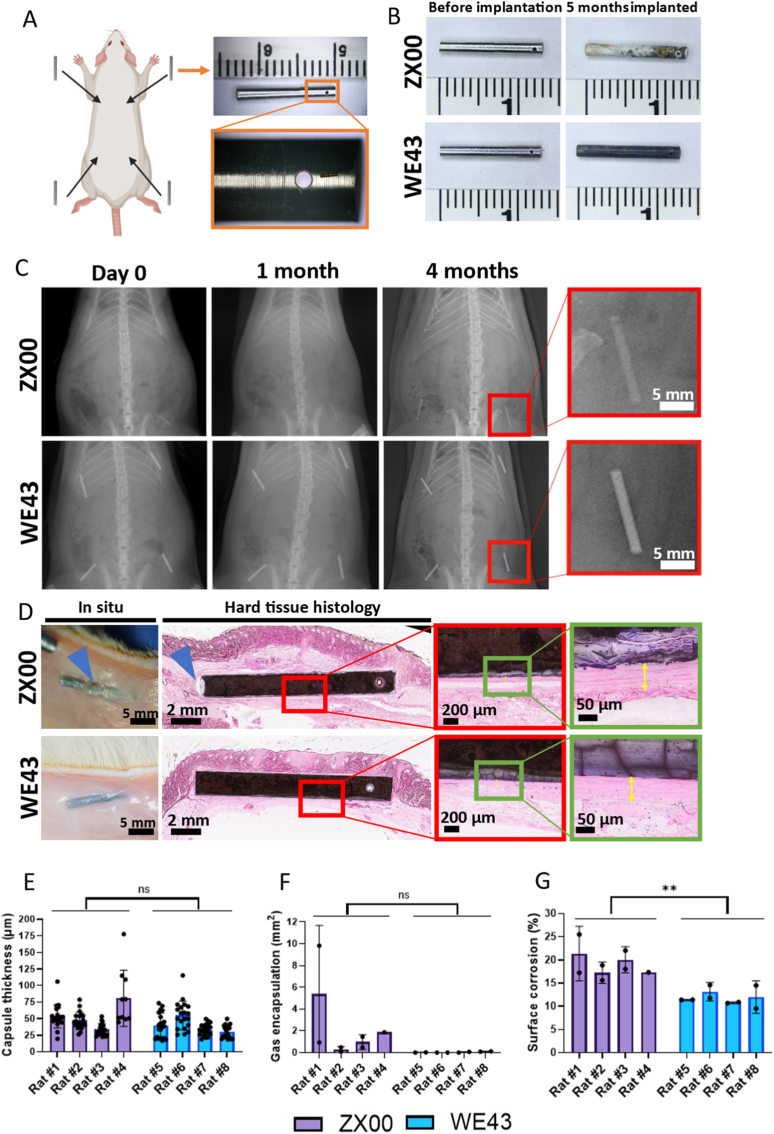
Subcutaneous implantation of ZX00 and WE43 rods in male Sprague–Dawley rats. (A) Dorsal implantation of four rods per animal, secured by a laser-drilled hole. (B) Representative images of rods before implantation and after 5 months *in vivo*. (C) X-ray follow-up imaging during the implantation period. (D) *In situ* appearance of implants after skin opening and corresponding histological sections (toluidine blue and pararosaniline staining). Blue arrows indicate hydrogen gas inclusions. Yellow arrows indicate the fibrous capsule surrounding the implant. (E) Fibrous capsule thickness. (F) Area of gas accumulation surrounding the implant. (G) Percentage of surface corrosion. Data are presented as mean ± SD. ns = not significant, ∗∗*p* < 0.01. (For interpretation of the references to colour in this figure legend, the reader is referred to the Web version of this article.)

The *in vivo* biocompatibility of ZX00 and WE43 alloys was evaluated through dorsal subcutaneous implantation of rods (n = 4 animals per alloy). Implant positioning was secured with sutures threaded through a laser-drilled hole at one end of each rod (Fig. 4A). All implantation sites healed normally, with no signs of inflammation or infection. Body weights increased physiologically according to age and strain (Supplementary Fig. 8). After 5 months, the explanted materials showed distinct appearances (Fig. 4B). WE43 was covered by a homogeneous black corrosion layer with only mild attachment to surrounding skin tissue, whereas ZX00 developed a rough white corrosion layer that adhered firmly to subcutaneous tissue.

X-ray images revealed no significant degradation or gas formation in either group (Fig. 4C). However, consistent differences in radiopacity between the materials were observed throughout the implantation period, attributable to differences in alloy composition.

To assess the implant–tissue interface, longitudinal sections were prepared using a specialized hard-tissue embedding protocol that preserved tissue morphology, including the entire metallic implant (Fig. 4D).

The mean thickness of the fibrous capsule surrounding the implants did not differ significantly between ZX00-and WE43-implanted animals (Fig. 4E). ZX00 showed a tendency of more gas encapsulations (Fig. 4F), however, this finding was not statistically significant. Signs of degradation were evident in both groups, but the measured area of corrosion (Fig. 4G) was significantly greater in the ZX00 group.

### 3.7 Mechanical testing and μCT of implanted ZX00 and WE43 rods

**Fig. 5:**
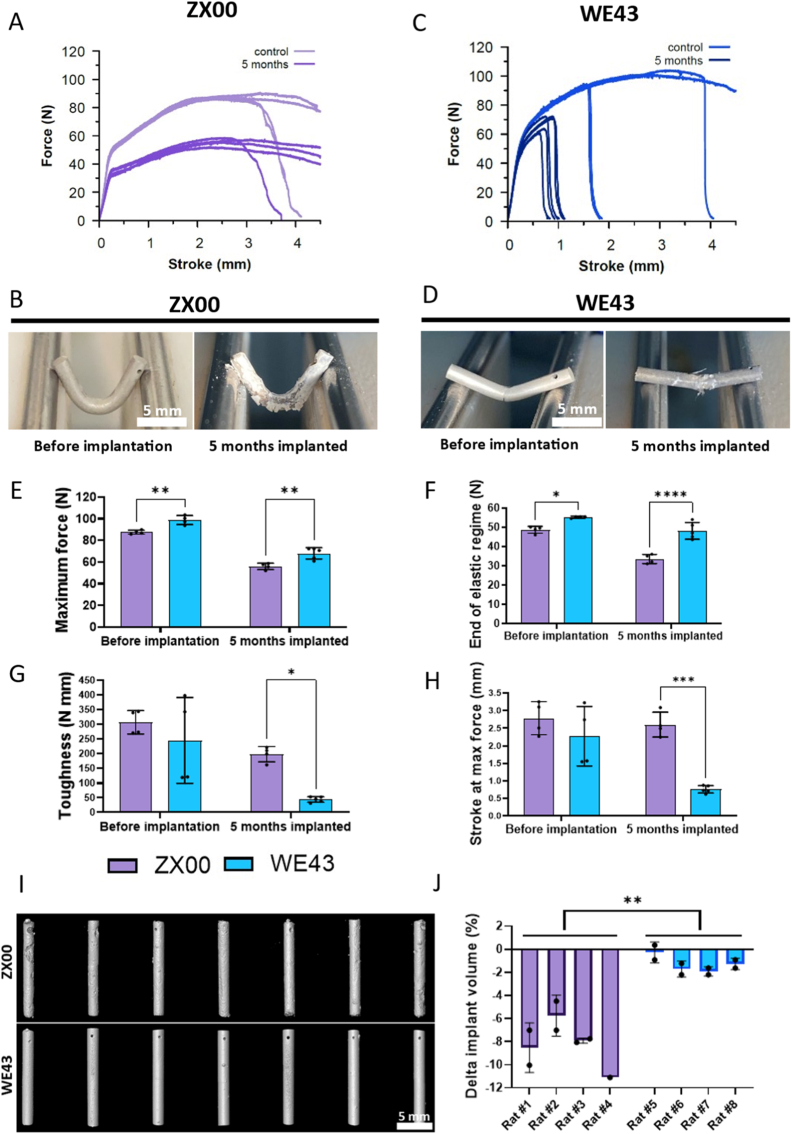
Three-point bending tests and μCT analysis of subcutaneously implanted ZX00 and WE43 rods. (A, C) Force–stroke curves of ZX00 and WE43 pins before implantation (controls) and after 5 months *in vivo*. (B, D) Representative images of pins immediately after bending tests. (E) Maximum force, (F) stroke at maximum force, (G) toughness, and (H) end of the elastic regime (force at onset of plastic deformation). (I) Representative μCT images of rods after 5 months of implantation. (J) Percentage change in implant volume after 5 months of implantation. Data are presented as individual values with mean ± SD. ∗*p* < 0.05, ∗∗*p* < 0.01, ∗∗∗*p* < 0.001, ∗∗∗∗*p* < 0.0001.

Consistent with the *in vitro* degradation studies (Fig. 3), ZX00 alloys exhibited significantly higher ductility and toughness than WE43 after 5 months of implantation (Fig. 5A–D). Maximum force decreased in both alloys compared with non-implanted controls. WE43 maintained significantly higher maximum force than ZX00 (Fig. 5E; *p* < 0.01) although the samples (just like in the *in vitro* study) fractured at low deflection values. Similarly, the transition from elastic to plastic deformation occurred at higher forces in WE43 both before (*p* < 0.01) and after implantation (*p* < 0.0001) (Fig. 5H).

Conversely, deformability, expressed as stroke at maximum force, was significantly better preserved in ZX00 (Fig. 5H; *p* < 0.001) also resulting in a higher toughness (Fig. 5G). High-resolution μCT analysis further confirmed the degradation profile of both alloys, revealing greater volume loss in ZX00 (Fig. 5I–J).

### 3.8 Surface corrosion of *in vitro* and *in vivo* degraded rod samples

**Fig. 6:**
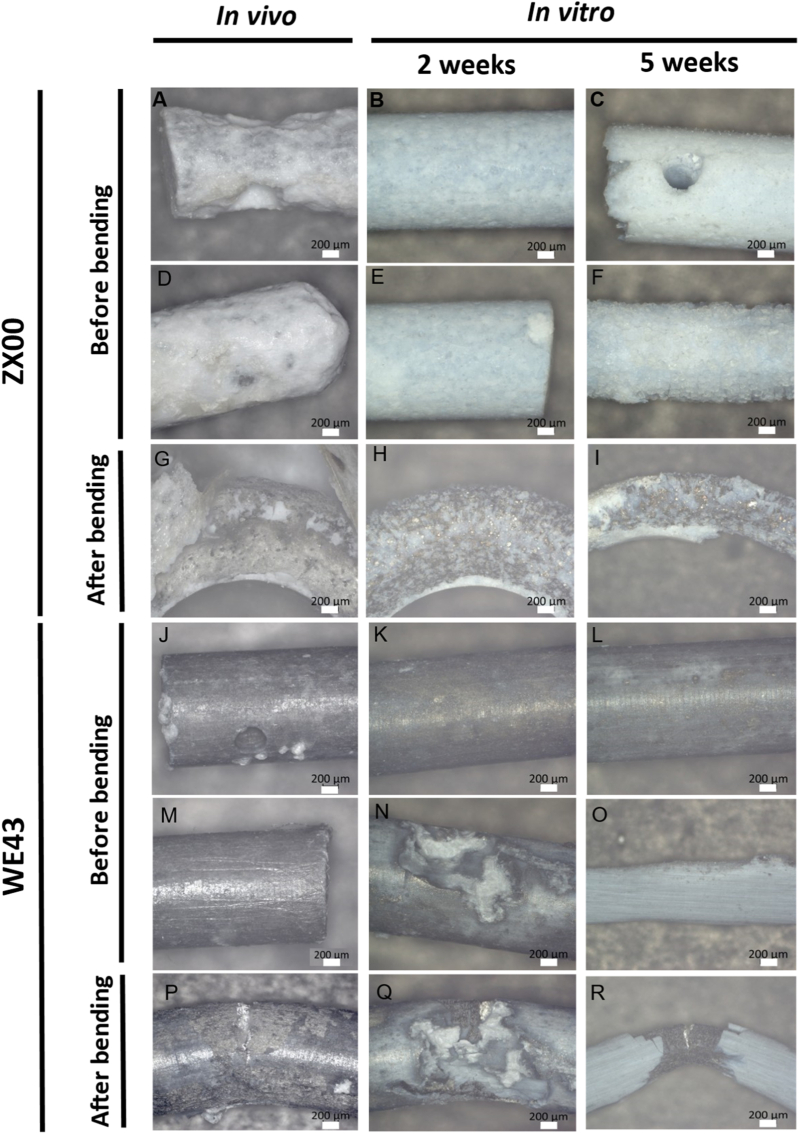
Light microscopy images of Mg pins after *in vivo* and *in vitro* degradation as well as after performing the bending tests. (A, D) Ends of ZX00 pins implanted for 5 months s.c. in rats. (B, E) ZX00 pins after 2 weeks of *in vitro* degradation in SBF. (C, F) ZX00 after 5 weeks *in vitro* degradation. (G) image of the highest bent region of an *in vivo* degraded ZX00 sample after the bending test. (H) ZX00 pin after 2 weeks *in vitro* degradation and bending test. (I) ZX00 pin after 5 weeks in SBF and subsequent bending. (J, M) WE43 pins after i*n vivo* degradation. (K, N) WE43 surfaces after 2 weeks in SBF. (L, O) WE43 pins after 5 weeks *in vitro* degradation. (P) WE43 pin fractured in the bending test after *in vivo* degradation. (Q) WE43 pin after 2 weeks *in vitro* degradation and bending test. (R) surface of WE43 after 5 weeks *in vitro* degradation and bending.

The surfaces of Mg pins after degradation were investigated by light microscopy. In case of ZX00 the images (Fig. 6A–I) revealed a brittle, white layer of degradation byproducts on the whole surface after *in vivo* as well as *in vitro* degradation. The metallic surface beneath was not observable this way.

The uniform loss of cross-sectional area without the formation of pits can also be seen in the cross-sections presented in Supplementary Fig. 7A and 7D. WE43 pins showed a different appearance. However, also in these samples the metallic surface was not visible but a brittle, this time black degradation surface layer (Fig. 6J–N).

An exception can be seen in Fig. 6O where most of the degradation layer fell off during testing and drying. Fig. 6N shows that WE43 is prone to localized corrosion and deep pits and holes on the surface evolve during degradation. Images of the surfaces after bending were also taken at the center positions of the pins where deformation was highest. They can be seen in Fig. 6G–I for ZX00 and Fig. 6P–R for WE43. For both alloys, the highly brittle degradation layer fell off during the bending tests.

As already reported in sections 3.5, 3.7, ZX00 was highly ductile after degradation. Accordingly, no signs of cracks were observed. A contrary observation was made for WE43. The samples fractured during the tests at the highest bent part or at a position of localized corrosion nearby. Representative fracture surfaces can be found in Supplementary Figs. 7B, C, E & F. The samples frequently fracture at points where pitting has reduced the cross-sectional area to a roughly semicircular shape. Such deep notches not only reduce the cross-sectional area but also lead to significant stress concentration.

### 3.9 Histocompatibility of implants

**Fig. 7:**
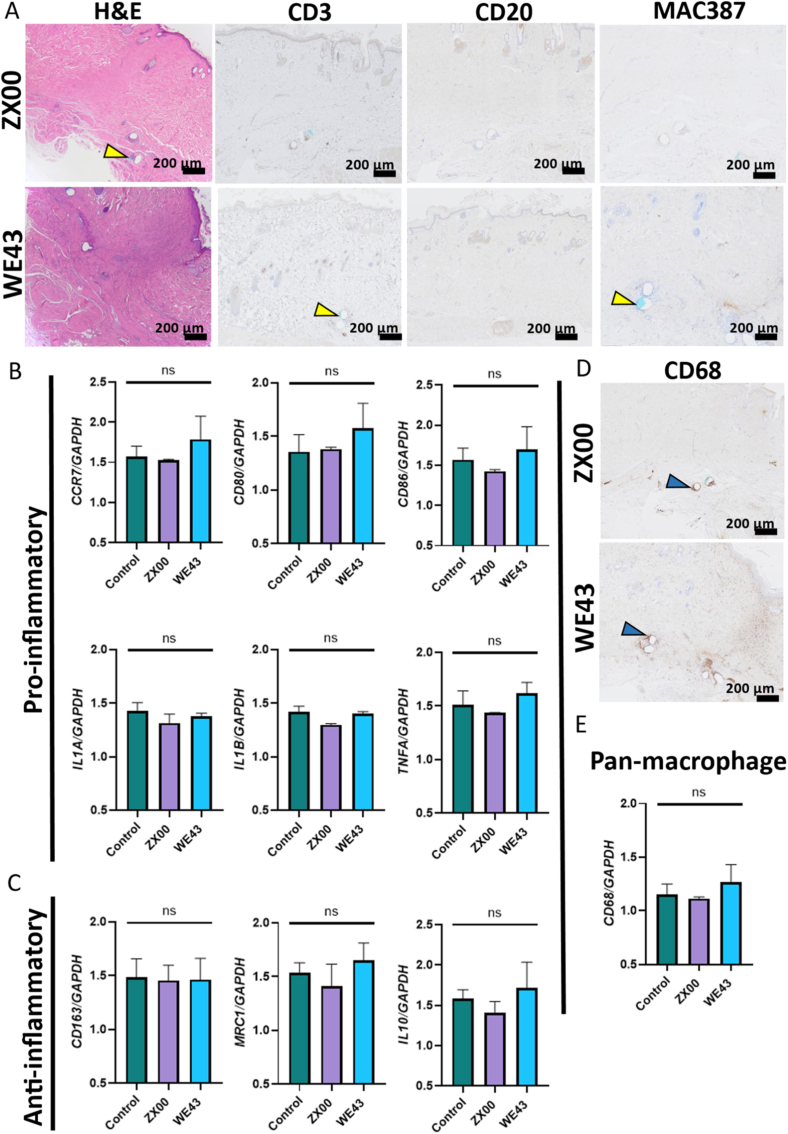
Local inflammatory response after 5 months of subcutaneous implantation of ZX00 and WE43. (A) Representative images of H&E staining and immunohistochemical staining (CD3, CD20, MAC387) of skin tissue adjacent to implants. Yellow arrows show remnants of suture material indicating the proximity of the implanted material (B) qPCR analysis of proinflammatory macrophage and cytokine markers (CCR7, CD80, CD86, IL-1α, IL-1β, and TNFα). (C) qPCR analysis of anti-inflammatory markers (CD163, IL-10, and MRC1). (D) Immunohistochemical stainings for CD68. Blue arrows indicate positively stained cells. (E) qPCR analysis of the pan-macrophage marker CD68 in implant-adjacent skin tissue. Data are presented as mean ± SD. ns = not significant. (For interpretation of the references to colour in this figure legend, the reader is referred to the Web version of this article.)

Immunohistochemical staining of skin tissue adjacent to the implants revealed moderate macrophage (CD68) infiltration and very mild infiltration of T-cells (CD3) and B-cells (CD20) in both implantation groups. These results were consistent with those of H&E staining, which showed no evidence of necrosis, granuloma formation, or foreign body giant cells (Fig. 7A).

The absence of active inflammation was further supported by the lack of MAC387^+^ cells. Gene expression analysis of implant-adjacent versus remote control skin tissue (Fig. 7B, C, E) corroborated these findings. No significant differences were detected in the expression levels of proinflammatory markers (CCR7, CD80, CD86, IL-1α, IL-1β, and TNFα), anti-inflammatory markers (CD163, IL-10, and MRC-1), or the pan-macrophage marker CD68. Together, these findings indicate the absence of severe inflammatory reactions at the implant–tissue interface or in surrounding tissue.

### 3.10 Toxicological evaluation

Histopathological scoring of the brain, heart, lungs, liver, kidneys, and spleen revealed no toxicopathological lesions in any of the implant groups (Supplementary Table 5). The microscopic findings recorded were mostly of minor grades and are amongst those spontaneous background findings seen in healthy rats at this age.

**Fig. 8:**
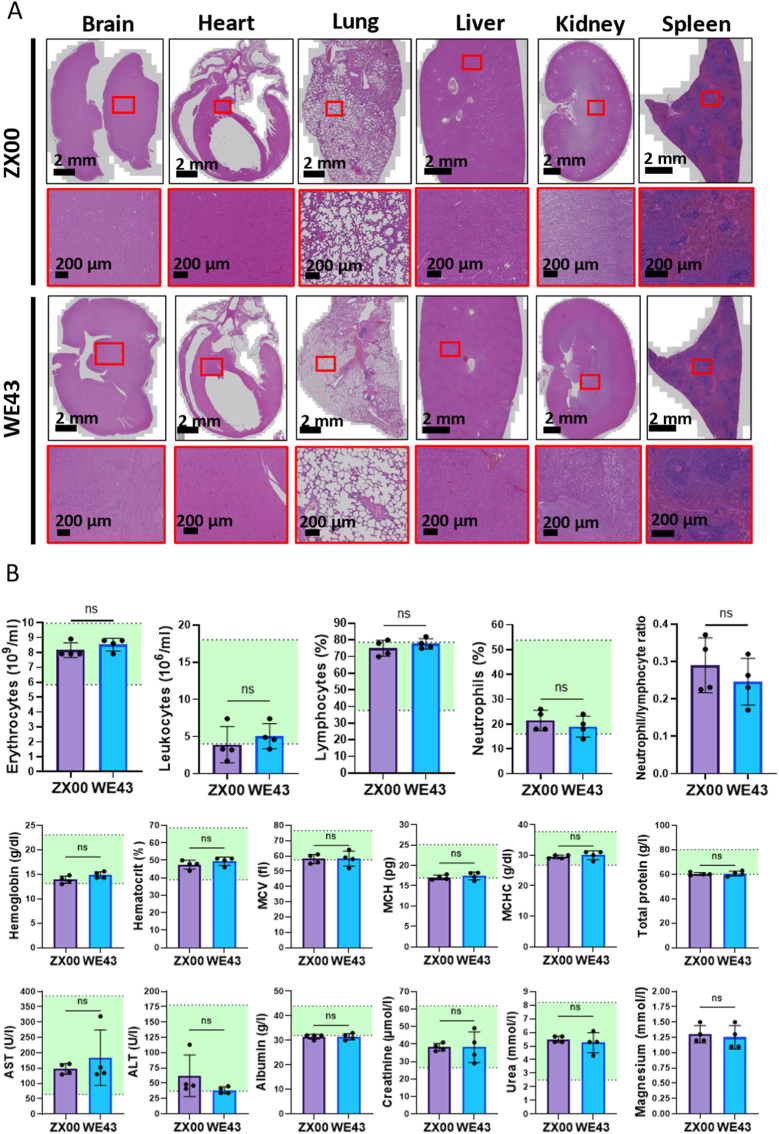
Histopathological and systemic toxicity assessments, hemograms, and toxicological blood panel after 5 months of subcutaneous implantation. (A) Representative H&E-stained sections of selected parenchymal organs. (B) Selected parameters from hemograms and blood chemistry analyses. Reference ranges for healthy male Sprague–Dawley rats are indicated in green. Data are shown as individual values with mean ± SD. ns = not significant. (For interpretation of the references to colour in this figure legend, the reader is referred to the Web version of this article.)

Representative images of H&E-stained sections of the above-mentioned parenchymatous organs are shown in Fig. 8A. These results indicate the absence of structural or cellular abnormalities.

Furthermore, comprehensive blood analyses, including hemograms, blood smears, and blood chemistry, were performed to detect signs of systemic toxicity, immune alterations, or organ dysfunction. The results showed no abnormalities or deviations from the reference values provided by the animal supplier (Charles River Laboratories) for male Sprague–Dawley rats, indicating no evidence of systemic toxicity.

In addition, the neutrophil to lymphocyte ratio showed no significant differences between groups, confirming the absence of a systemic inflammatory response and supporting implant biocompatibility. Finally, magnesium plasma levels also showed no statistically significant differences between the groups (Fig. 8B).

These findings suggest that material degradation and associated ion release did not result in detectable systemic alterations and are likely effectively locally buffered under the investigated conditions.

## 4 Discussion

Mg alloys are promising candidates to replace permanent implants in orthopedics and cardiovascular medicine because of their biodegradability and excellent biocompatibility. Clinical trials support this potential, such as the BIOMAG-I study (NCT04157153), in which a Mg-based stent showed no scaffold thrombosis and only one target lesion failure among 116 patients at 6 months. Additionally, the large BIOMAG-II trial (NCT05540223) is currently ongoing [39]. Earlier BIOSOLVE I–IV trials also evaluated Biotronik's REE-containing Mg stents, which are comparable in composition to the WE43 alloy tested here. Although these results underscore translational feasibility, the long-term biological effects of both light and heavy REEs released during degradation remain uncertain [8]. For example, a preclinical study with WZ21 implants (Mg–2Y–1Zn–0.25Ca–0.25Mn) demonstrated Y accumulation in cortical bone [40], raising concerns for alloys such as WE43 with even higher Y content. Cytotoxic effects of several rare-earth elements have been described before. Brouziotis et al. [41] highlighted ROS production and DNA damage as common causes for REE-associated cytotoxicity. These might also be the underlying mechanisms for the lower viability observed in our cells treated with our ytterbium-containing Mg alloy ZX00+. Rare-earth elements are incorporated into magnesium alloys to improve corrosion resistance and the overall mechanical properties simultaneously, thus also the structural stability of Mg-based medical implants during degradation. However, despite these advantages, considerations regarding the long-term biological fate of rare-earth elements, potential accumulation and cytotoxicity in case of elevated local concentrations, and regulatory or sustainability aspects have prompted increasing interest in the development of rare-earth-element-free magnesium alloy systems [8,42,43].

To advance the development of Mg alloys independent of REEs, we comprehensively evaluated the REE-free alloy ZX00 (Mg–0.6Zn–0.5Ca) against the established REE-containing WE43 alloy through *in vitro* and *in vivo* studies, focusing on biocompatibility, inflammatory responses, histopathology, systemic toxicological profile, and mechanical performance. In this context, also a variant containing a small addition of ytterbium (ZX00+) was developed to reduce crack susceptibility during processing [31]. The inclusion of ZX00+ in this study allows assessment of whether such a minor REE addition influences biocompatibility and microstructural characteristics, particularly in comparison to the base ZX00 alloy.

Mg–Zn alloys are generally well tolerated, though their corrosion resistance is limited by Zn-rich precipitates and Zn oxide layers [44]. The addition of Ca, an essential element in the human body, can enhance corrosion resistance when maintained below 1 wt% [45] especially if the zinc content is kept low simultaneously [46]. This alloying strategy enables the formulation of safer bioresorbable materials by eliminating REEs while optimizing degradation behavior, thereby supporting the clinical translation of Mg-based implants. Further, to rule out the risk of tissue calcification (particularly in our intended potential application as annuloplasty ring) the Ca content is kept very low (0.5 wt%), corresponding to approximately 4 mg per implant for one ZX00 annuloplasty ring. Considering this minimal amount and the continuous high cardiac blood flow ensuring substantial dilution, the risk of local calcification is expected to be negligible.

Our biocompatibility studies revealed that both WE43 and ZX00 supported high cell viability when tested with 50% diluted extracts and pH adjustment. However, under harsher conditions (100% extracts with or without byproduct exclusion), WE43 caused a significant reduction in fibroblast viability, suggesting that a concentration-dependent cytotoxic influence of REEs might be possible. Both WE43 and ZX00 demonstrated hemocompatibility, and the acceptable hemolysis levels observed for WE43 are consistent with earlier findings by Zhen et al. [47]. To our knowledge, as hemolysis and coagulation data for ZX00 have not been published before, our results offer initial insight into its blood-material interactions.

The innate and adaptive immune responses are critical determinants of Mg implant performance. In vascular stent applications, the degree of vessel injury during implantation strongly influences inflammation and remodeling [25]. Acute inflammation typically involves infiltration of plasma cells, monocytes, neutrophils, and lymphocytes, followed by macrophage polarization toward either the proinflammatory M1 or anti-inflammatory M2 phenotype, which regulate pathogen clearance and tissue repair. Healing then progresses through fibroblast activation, angiogenesis, and tissue remodeling [48]. Wu et al. reported prominent macrophage polarization toward the anti-inflammatory M2 subtype when culturing with extracts of the “JiaoDa BioMg” (JDBM: Mg–2.1Nd–0.2Zn–0.5Zr) alloy, developed for vascular stent applications [49]. In our study, ZX00 induced a mild anti-inflammatory response in macrophages derived from PBMCs, with gene expression analyses showing mild polarization toward M2 macrophages *In vivo*, expression of pro- and anti-inflammatory markers remained balanced, corroborated by histological analysis. These results suggest that ZX00 elicits only a limited inflammatory response, supporting its biocompatibility in soft tissue. Furthermore, macrophage polarization, assessed by the CCR7/CD163 ratio *in vitro*, showed no significant differences between groups, indicating that neither alloy preferentially drove pro-inflammatory (M1) polarization. Instead, all conditions favored a predominantly M2 like, anti-inflammatory phenotype, suggesting a generally immunomodulatory rather than inflammatory response.

In physiological environments, the biodegradation of Mg alloys is often characterized by rapid corrosion and localized pitting. Such pitting can compromise the structural integrity of Mg-based implants, leading to stress concentrations during mechanical loading and premature mechanical failure before healing is completed. This has also been shown recently by Ulugun et al. and Raguraman et al., who further examined the microstructural features governing the pitting behavior. This has also been shown recently by Ulugun et al. and Raguraman et al. who furthermore investigated the microstructural details responsible for the pitting behavior in detail [50,51]. Achieving high toughness and reliable mechanical performance is essential for a safe clinical application. In our three-point bending tests, non-degraded WE43 exhibited superior mechanical strength owing to its higher alloying content (∼7 wt% vs. ∼1 wt% in ZX00) and the formation of numerous precipitates. However, ductility (stroke at maximum force) and toughness of WE43 were significantly lower and showed high variance. Furthermore, our results on the mechanical behavior after degradation show clear advantages of ZX00. While the load-bearing capacity during the ongoing degradation depends primarily on the macroscopic loss of mass (as can be seen in Fig. 3K) and appears less influenced by the specific degradation mechanisms or microstructural features, the behavior of ductility and consequently toughness is more complex and governed by local degradation characteristics. WE43 exhibited a pronounced loss of ductility, whereas ZX00 maintained its ductility despite higher degradation rates and volume loss both *in vitro* and *in vivo*. Finally, ZX00 also showed higher toughness and overall robustness of mechanical behavior after implantation and *in vivo* degradation.

The reason lies in the more uniform corrosion behavior of ZX00 compared to WE43 which promotes gradual, controlled material loss with reduced damage localization, as evidenced in Fig. 6 and Supplementary Fig. 7. In case of WE43, localized corrosion leads to stress concentrations, reduced toughness, and a pronounced loss of ductility, ultimately resulting in premature failure.

The more uniform corrosion behavior observed in ZX00 can be attributed to the smaller micro-galvanic interaction between its precipitates and the Mg matrix. As shown previously, ZX00 contains small Mg_2_Ca precipitates which are even less noble than the Mg matrix [28]. WE43, which contains a higher overall alloying content, exhibits a correspondingly greater amount of precipitates (as shown in Supplementary Fig. 1). In addition to having a higher mass fraction, these precipitates are now also more noble than the Mg matrix due to their chemical composition [52]. Although ZX00 shows a pronounced texture and a mixture of deformed and recrystallized grains, the electrochemical results suggest that the precipitate effect in WE43 alloy exceeds the texture effect of ZX00. In total, WE43 shows a slightly higher activity due to the rare-earth-rich precipitates, which act as cathodic sites and promote micro-galvanic interactions.

Thus, ZX00 emerges as a promising candidate for applications requiring stability and ductility during the service life of the implant, such as in cardiovascular medicine. More precisely, cardiac repair devices such as annuloplasty rings could be suitable implant applications.

In the subcutaneous implantation model, all animals survived to the planned endpoint without signs of severe local inflammation or toxicity. Wound healing progressed without complications, and all animals exhibited normal physiological weight gain (Supplementary Fig. 8). As expected, *in vivo* degradation of Mg alloys generated hydrogen gas, a potential complication in both orthopedic and cardiovascular applications [53]. While gas pockets may delay tissue integration in bone implants [54,55] or interfere with blood flow in stents if excessive [56,57] the hydrogen evolution observed here remained within a biologically tolerable range. To our knowledge, a universally accepted physiological threshold for acceptable hydrogen evolution from Mg-based implants has not been clearly defined. Consequently, biological tolerability is typically assessed using qualitative and semi-quantitative indicators, such as the persistence of gas formation and the associated tissue response. In agreement with prior studies, moderate and transient hydrogen evolution has generally been reported to be well tolerated *in vivo* for biodegradable Mg implants [58,59]. Radiographs confirmed implant integrity without breakage and showed no evidence of excessive gas accumulation. A unique μCT-guided histological approach that has not been used before in subcutaneous implantation models revealed greater overall corrosion of ZX00 than WE43. However, no significant differences were observed in the amount of fibrous tissue surrounding the alloys (Fig. 4E).

Pathological analysis of H&E-stained sections of brain, heart, lungs, liver, spleen, and kidneys revealed no evidence of systemic toxicity. The safety of both alloys was further supported by hemograms and analysis of circulating toxicological parameters. For certain parameters, such as leukocyte counts and erythrocyte indices, individual animals in both groups exhibited values below the physiological range (according to reference values provided by Charles River Laboratories). However, these deviations did not result in any statistically significant differences between the two implantation groups. Taken together with the detailed histopathological analyses, these findings indicate that no animals in either group exhibited signs of systemic toxicity.

In summary, this study provides a comprehensive characterization of Mg–0.6Zn–0.5Ca (ZX00), demonstrating preserved toughness after 5 months of subcutaneous degradation, minimal inflammation, limited gas formation, absence of systemic toxicity, and overall excellent performance. To the best of our knowledge, no previous study has combined such detailed assessments of mechanical properties, tissue-implant interactions, systemic biocompatibility, toxicological blood parameters, and histopathological organ responses for a REE-free ZX00 alloy. Although preclinical research on ZX00 has so far focused primarily on orthopedic applications, with promising outcomes in both small and large animal models [19,20,30], clinical evidence is only beginning to emerge. Labmayr et al. recently reported long-term sheep implantations and a pilot clinical study of malleolar fracture fixation in 20 patients, showing nearly complete implant resorption after 2.5 years without complications [60]. They further demonstrated that ZX00 exhibits favorable mechanical stability and degradation behavior in less load-bearing orthopedic applications, supporting its potential as a viable alternative to WE43 in indications where extreme mechanical strength is not required. Our findings underscore ZX00 as a safe and reliable material with strong translational potential, extending its potential beyond orthopedics to cardiovascular and regenerative medicine. Accordingly, the material is intended for use as a valvular cardiac repair device, specifically a mitral annuloplasty ring. Therefore, this manuscript emphasizes biocompatibility, mechanical integrity, and controlled degradation compatible with cardiac valve repair requirements.

## 5 Limitations

The authors note that the comparative assessment presented here is subject to some limitations. WE43 and ZX00 differ in alloy chemistry, processing, and resulting microstructures, which can strongly influence corrosion behavior and degradation morphology. Furthermore, these outcomes depend on the used corrosive liquid and the implantation site for *in vitro* and *in vivo* tests, respectively. The cross-section and appearance of the surface, in turn, determine the mechanical properties presented here. The results therefore cannot be extrapolated to all manufacturing processes and environmental variables.

Further, in the present work, degradation and hemocompatibility were primarily evaluated using static *in vitro* assays. While this approach is appropriate for an initial material assessment, it does not fully capture the dynamic mechanical loading and complex hemodynamic conditions of the cardiac environment. Accordingly, future studies are investigating material degradation and thrombogenicity under dynamic and physiologically relevant conditions to further characterize *in vivo* performance.

## 6 Conclusion

A self-designed, REE-free Mg–Zn–Ca alloy (Mg–0.6Zn–0.5Ca; ZX00) was utilized, demonstrating excellent mechanical toughness, biocompatibility, and low immunogenicity. Although Mg alloys have been explored as biomaterials for decades, concerns persist regarding the long-term biocompatibility of REE-containing systems. In this context, the REE-free alloy ZX00 exhibited significantly better preservation of ductility than the widely used WE43 alloy in both *in vitro* and *in vivo* evaluations. Furthermore, ZX00 showed hemocompatibility and *in vivo* toxicological safety comparable to WE43. Collectively, these findings identify ZX00 as a promising candidate for cardiovascular applications such as cardiac annuloplasty devices, offering robust performance while eliminating the need for REE alloying.

## Declaration of AI and AI-assisted technologies in the writing process

Editorial assistance for grammar and language refinement was provided using an AI-based tool (GPT-4o; OpenAI, San Francisco, CA, USA). The authors reviewed and edited all content and take full responsibility for the final manuscript.

## Funding

This work was supported by the state of Lower Austria (NÖ Wirtschafts-und Tourismusfonds, WST3-F-5030665/009-2020). The funding agency was not involved in study design and collection, analysis and interpretation of data.

## CRediT authorship contribution statement

**Lukas Weber:** Data curation, Formal analysis, Investigation, Validation, Visualization, Writing – original draft, Writing – review & editing. **Jelena Horky:** Data curation, Formal analysis, Funding acquisition, Investigation, Methodology, Resources, Validation, Visualization, Writing – original draft, Writing – review & editing. **Christopher Riedmüller:** Data curation, Formal analysis, Investigation, Validation, Visualization, Writing – review & editing. **Carina Kampleitner:** Data curation, Formal analysis, Investigation, Validation, Visualization, Writing – review & editing. **Eylem Acar:** Data curation, Formal analysis, Investigation. **Mariangela Fedel:** Funding acquisition, Writing – review & editing. **Claudia Höchsmann:** Data curation, Formal analysis, Investigation, Validation, Writing – review & editing. **Magdalena Eskinja:** Data curation, Formal analysis, Investigation, Methodology, Visualization, Writing – original draft, Writing – review & editing. **Stefan Mitsche:** Data curation, Formal analysis, Investigation, Methodology, Visualization, Writing – original draft, Writing – review & editing. **Bernhard Mingler:** Conceptualization, Funding acquisition, Methodology, Project administration, Resources, Writing – review & editing. **Laszlo Sajti:** Funding acquisition, Writing – review & editing. **Gregor Mori:** Investigation, Methodology, Supervision, Writing – original draft, Writing – review & editing. **Manfred Bammer:** Conceptualization, Funding acquisition, Project administration, Resources, Supervision, Writing – review & editing. **Ingrid Walter:** Formal analysis, Methodology, Resources, Validation, Writing – review & editing. **Stefan Tangl:** Methodology, Resources, Writing – review & editing. **Helga Bergmeister:** Conceptualization, Funding acquisition, Methodology, Project administration, Resources, Supervision, Validation, Writing – review & editing. **Bruno K. Podesser:** Conceptualization, Funding acquisition, Project administration, Supervision, Writing – review & editing. **Marjan Enayati:** Conceptualization, Data curation, Formal analysis, Investigation, Methodology, Project administration, Resources, Supervision, Validation, Visualization, Writing – original draft, Writing – review & editing.

## Declaration of competing interest

The authors declare that they have no known competing financial interests or personal relationships that could have appeared to influence the work reported in this paper.

## Data Availability

Data will be made available on request.

## References

[1] Li X., Liu X., Wu S., Yeung K.W.K., Zheng Y., Chu P.K. (2016). Design of magnesium alloys with controllable degradation for biomedical implants: from bulk to surface. Acta Biomater..

[2] Esmaily M., Svensson J.E., Fajardo S., Birbilis N., Frankel G.S., Virtanen S., Arrabal R., Thomas S., Johansson L.G. (2017). Fundamentals and advances in magnesium alloy corrosion. Prog. Mater. Sci..

[3] Agarwal S., Curtin J., Duffy B., Jaiswal S. (2016). Biodegradable magnesium alloys for orthopaedic applications: a review on corrosion, biocompatibility and surface modifications. Mater. Sci. Eng. C.

[4] Zhang Z.-Q., Yang Y.-X., Li J.-A., Zeng R.-C., Guan S.-K. (2021). Advances in coatings on magnesium alloys for cardiovascular stents – a review. Bioact. Mater..

[5] Zhang T., Wang W., Liu J., Wang L., Tang Y., Wang K. (2022). A review on magnesium alloys for biomedical applications. Front. Bioeng. Biotechnol..

[6] Lin B., Zhong M., Zheng C., Cao L., Wang D., Wang L., Liang J., Cao B. (2015). Preparation and characterization of dopamine-induced biomimetic hydroxyapatite coatings on the AZ31 magnesium alloy. Surf. Coat. Technol..

[7] Hernández L., González J.E., Barranco V., Veranes-Pantoja Y., Galván J.C., Gattorno G.R. (2022). Biomimetic hydroxyapatite (HAp) coatings on pure Mg and their physiological corrosion behavior. Ceram. Int..

[8] Weng W., Biesiekierski A., Li Y., Dargusch M., Wen C. (2021). A review of the physiological impact of rare earth elements and their uses in biomedical Mg alloys. Acta Biomater..

[9] Nicolas J., Pivato C.A., Chiarito M., Beerkens F., Cao D., Mehran R. (2023). Evolution of drug-eluting coronary stents: a back-and-forth journey from the bench to bedside. Cardiovasc. Res..

[10] Yuen C.K., Ip W.Y. (2010). Theoretical risk assessment of magnesium alloys as degradable biomedical implants. Acta Biomater..

[11] Yang M., Chen C., Wang D., Shao Y., Zhou W., Shuai C., Yang Y., Ning X. (2024). Biomedical rare-earth magnesium alloy: current status and future prospects. J. Magnesium Alloys.

[12] Wu X., Liu J., Yang Y., Bai J., Shuai C., Buhagiar J., Ning X. (2024). Laser powder bed fusion of biodegradable magnesium alloys: process, microstructure and properties. Int. J. Extrem. Manuf..

[13] Jang Y., Tan Z., Jurey C., Xu Z., Dong Z., Collins B., Yun Y., Sankar J. (2015). Understanding corrosion behavior of Mg–Zn–Ca alloys from subcutaneous mouse model: effect of Zn element concentration and plasma electrolytic oxidation. Mater. Sci. Eng. C.

[14] Chen L., Li J., Zhao D., Guo W., Wu S., Lü S. (2024). Development of rare-earth free Mg–Si–Zn–Cu alloy with outstanding mechanical properties and high thermal conductivity. Mater. Sci. Eng., A.

[15] Liu Y., Lu B., Cai Z. (2019). Recent progress on Mg- and Zn-Based alloys for biodegradable vascular stent applications. J. Nanomater..

[16] Mahto V.K., Singh A.K., Malik A. (2023). Surface modification techniques of magnesium-based alloys for implant applications. J. Coat. Technol. Res..

[17] Li Z., Gu X., Lou S., Zheng Y. (2008). The development of binary Mg–Ca alloys for use as biodegradable materials within bone. Biomaterials.

[18] Zhang B., Hou Y., Wang X., Wang Y., Geng L. (2011). Mechanical properties, degradation performance and cytotoxicity of Mg–Zn–Ca biomedical alloys with different compositions. Mater. Sci. Eng. C.

[19] Holweg P., Berger L., Cihova M., Donohue N., Clement B., Schwarze U., Sommer N.G., Hohenberger G., van den Beucken J.J.J.P., Seibert F., Leithner A., Löffler J.F., Weinberg A.-M. (2020). A lean magnesium–zinc–calcium alloy ZX00 used for bone fracture stabilization in a large growing-animal model. Acta Biomater..

[20] Kopp A., Fischer H., Soares A.P., Schmidt-Bleek K., Leber C., Kreiker H., Duda G., Kröger N., van Gaalen K., Hanken H., Jung O., Smeets R., Heiland M., Rendenbach C. (2023). Long-term *in vivo* observations show biocompatibility and performance of ZX00 magnesium screws surface-modified by plasma-electrolytic oxidation in göttingen miniature pigs. Acta Biomater..

[21] Lan W., Li J., Lv Z., Liu S., Liang Z., Huang D., Wei X., Chen W. (2024). *In vitro* corrosion and cytocompatibility of Mg-Zn-Ca alloys coated with FHA. Colloids Surf. B Biointerfaces.

[22] Waksman R., Pakala R., Kuchulakanti P.K., Baffour R., Hellinga D., Seabron R., Tio F.O., Wittchow E., Hartwig S., Harder C., Rohde R., Heublein B., Andreae A., Waldmann K.-H., Haverich A. (2006). Safety and efficacy of bioabsorbable magnesium alloy stents in porcine coronary arteries, Catheter. Cardiovasc. Interv..

[23] Zhu J., Zhang X., Niu J., Shi Y., Zhu Z., Dai D., Chen C., Pei J., Yuan G., Zhang R. (2021). Biosafety and efficacy evaluation of a biodegradable magnesium-based drug-eluting stent in porcine coronary artery. Sci. Rep..

[24] Jedrzejczyk J.H., Carlson Hanse L., Javadian S., Skov S.N., Hasenkam J.M., Thørnild M.J. (2022). Mitral annular forces and their potential impact on Annuloplasty ring selection. Front. Cardiovasc. Med..

[25] Ma J., Zhao N., Betts L., Zhu D. (2016). Bio-Adaption between magnesium alloy stent and the blood vessel: a review. J. Mater. Sci. Technol..

[26] Horky J., Ghaffar A., Werbach K., Mingler B., Pogatscher S., Schäublin R., Setman D., Uggowitzer P.J., Löffler J.F., Zehetbauer M.J. (2019). Exceptional strengthening of biodegradable mg-zn-ca alloys through high pressure torsion and subsequent heat treatment. Materials.

[27] Hofstetter J., Becker M., Martinelli E., Weinberg A.M., Mingler B., Kilian H., Pogatscher S., Uggowitzer P.J., Löffler J.F. (2014). High-Strength low-alloy (HSLA) mg–zn–ca alloys with excellent biodegradation performance. JOM.

[28] Horky J., Bryła K., Krystian M., Mozdzen G., Mingler B., Sajti L. (2021). Improving mechanical properties of lean Mg–Zn–Ca alloy for absorbable implants via Double Equal Channel Angular Pressing (D-ECAP). Mater. Sci. Eng., A.

[29] Martinez D.C., Dobkowska A., Marek R., Ćwieka H., Jaroszewicz J., Płociński T., Donik Č., Helmholz H., Luthringer-Feyerabend B., Zeller-Plumhoff B., Willumeit-Römer R., Święszkowski W. (2023). *In vitro and in vivo* degradation behavior of Mg-0.45Zn-0.45Ca (ZX00) screws for orthopedic applications. Bioact. Mater..

[30] Rahmati M., Stötzel S., Khassawna T.E., Iskhahova K., Florian Wieland D., Zeller Plumhoff B., Haugen H.J. (2021). Early osteoimmunomodulatory effects of magnesium–calcium–zinc alloys. J. Tissue Eng..

[31] Schiffl A., Mingler B. (2012). Magnesiumlegierung, WO2012003522A3. https://patents.google.com/patent/WO2012003522A3/de.

[32] Müller L., Müller F.A. (2006). Preparation of SBF with different HCO3- content and its influence on the composition of biomimetic apatites. Acta Biomater..

[33] Hertzberg R.W., Vinci R.P., Hertzberg J.L. (2012).

[34] Schneider C.A., Rasband W.S., Eliceiri K.W. (2012). NIH Image to ImageJ: 25 years of image analysis. Nat. Methods.

[35] Thaler B., Baik N., Hohensinner P.J., Baumgartner J., Panzenböck A., Stojkovic S., Demyanets S., Huk I., Rega-Kaun G., Kaun C., Prager M., Fischer M.B., Huber K., Speidl W.S., Parmer R.J., Miles L.A., Wojta J. (2019). Differential expression of Plg-RKT and its effects on migration of proinflammatory monocyte and macrophage subsets. Blood.

[36] Bankhead P., Loughrey M.B., Fernández J.A., Dombrowski Y., McArt D.G., Dunne P.D., McQuaid S., Gray R.T., Murray L.J., Coleman H.G., James J.A., Salto-Tellez M., Hamilton P.W. (2017). QuPath: open source software for digital pathology image analysis. Sci. Rep..

[37] Donath K., Breuner G. A method for the study of undecalcified bones and teeth with attached soft tissues. https://onlinelibrary.wiley.com/doi/10.1111/j.1600-0714.1982.tb00172.x.

[38] McWhorter F.Y., Wang T., Nguyen P., Chung T., Liu W.F. (2013). Modulation of macrophage phenotype by cell shape. Proc. Natl. Acad. Sci..

[39] Haude M., Wlodarczak A., van der Schaaf R.J., Torzewski J., Ferdinande B., Escaned J., Iglesias J.F., Bennett J., Toth G., Joner M., Toelg R., Wiemer M., Olivecrona G., Vermeersch P., Garcia-Garcia H.M., Waksman R. (2023). Safety and performance of the third-generation drug-eluting resorbable coronary magnesium scaffold system in the treatment of subjects with de novo coronary artery lesions: 6-month results of the prospective, multicenter BIOMAG-I first-in-human study. eClinicalMedicine.

[40] Amerstorfer F., Fischerauer S.F., Fischer L., Eichler J., Draxler J., Zitek A., Meischel M., Martinelli E., Kraus T., Hann S., Stanzl-Tschegg S.E., Uggowitzer P.J., Löffler J.F., Weinberg A.M., Prohaska T. (2016). Long-term *in vivo* degradation behavior and near-implant distribution of resorbed elements for magnesium alloys WZ21 and ZX50. Acta Biomater..

[41] Brouziotis A.A., Giarra A., Libralato G., Pagano G., Guida M., Trifuoggi M. (2022). Toxicity of rare earth elements: an overview on human health impact. Front. Environ. Sci..

[42] Li H., Wang P., Lin G., Huang J. (2021). The role of rare earth elements in biodegradable metals: a review. Acta Biomater..

[43] Chen J., Zhan J., Kolawole S.K., Tan L., Yang K., Wang J., Su X. (2022). Effects of different rare Earth elements on the degradation and mechanical properties of the ECAP extruded Mg alloys. Materials.

[44] Koç E., Kannan M.B., Ünal M., Candan E. (2015). Influence of zinc on the microstructure, mechanical properties and in vitro corrosion behavior of magnesium–zinc binary alloys. J. Alloys Compd..

[45] Zhang Y., Li J., Li J. (2017). Effects of calcium addition on phase characteristics and corrosion behaviors of Mg-2Zn-0.2Mn-xCa in simulated body fluid. J. Alloys Compd..

[46] Cihova M., Martinelli E., Schmutz P., Myrissa A., Schäublin R., Weinberg A.M., Uggowitzer P.J., Löffler J.F. (2019). The role of zinc in the biocorrosion behavior of resorbable Mg‒Zn‒Ca alloys. Acta Biomater..

[47] Zhen Z., Xi T., Zheng Y., Li L., Li L. (2014). *In Vitro* Study on mg–sn–mn alloy as biodegradable metals. J. Mater. Sci. Technol..

[48] Sun Q., Zhou Y., Zhang A., Wu J., Tan L., Guo S. (2024). The immunomodulatory effects and mechanisms of magnesium-containing implants in bone regeneration: a review. J. Magnesium Alloys.

[49] Wu J., Jin L., Tan J.-Y., Chen X.-F., Wang Q.-Q., Yuan G.-Y., Chen T.-X. (2021). The effects of a biodegradable Mg-based alloy on the function of VSMCs via immunoregulation of macrophages through Mg-induced responses. Ann. Transl. Med..

[50] Raguraman S., Connon M.L., Byrum C.N., Berlia R., Ivanovskaya V., Ulugun B., Eswarappa Prameela S., Guillory II R.J., Weihs T.P. (2025). Microstructure regulates early-stage corrosion behavior and systemic aluminum fate in biodegradable Mg–Al alloys: integrated in-vitro and in-vivo insights. Acta Biomater..

[51] Ulugun B., Raguraman S., Osei-Owusu N.B., Raj S., Ramirez C., Griebel A.J., Weihs T.P. (2025). Role of microstructure, corrosion, and pit geometry in governing strength and ductility loss in biodegradable magnesium alloy wires. J. Alloys Compd..

[52] Mraied H., Wang W., Cai W. (2019). Influence of chemical heterogeneity and microstructure on the corrosion resistance of biodegradable WE43 magnesium alloys. J. Mater. Chem. B.

[53] Guan S., Mei D., Wang J., Zhang Z., Du P., Bai L., Yan C., Li J., Wang J., Zhu S. (2023). Mg alloy cardio-/cerebrovascular scaffolds: developments and prospects. J. Magnesium Alloys.

[54] Noviana D., Paramitha D., Ulum M.F., Hermawan H. (2016). The effect of hydrogen gas evolution of magnesium implant on the postimplantation mortality of rats. J. Orthop. Transl..

[55] Chagnon M., Guy L.-G., Jackson N. (2019). Evaluation of magnesium-based medical devices in preclinical studies: challenges and points to consider. Toxicol. Pathol..

[56] Lin X., Saijilafu, Wu Xiexing, Wu Kang, Chen Jianquan, Tan Lili, Frank Witte, Huilin Yang, Diego Mantovani, Huan Zhou, Chunyong Liang, Qiang Yang, Ke L Yang, Yang (2023). Biodegradable Mg-based alloys: biological implications and restorative opportunities. Int. Mater. Rev..

[57] Tan J., Ramakrishna S. (2021). Applications of magnesium and its alloys: a review. Appl. Sci..

[58] Staiger M.P., Pietak A.M., Huadmai J., Dias G. (2006). Magnesium and its alloys as orthopedic biomaterials: a review. Biomaterials.

[59] Witte F., Ulrich H., Rudert M., Willbold E. (2007). Biodegradable magnesium scaffolds: part 1: appropriate inflammatory response. J. Biomed. Mater. Res..

[60] Labmayr V., Suljevic O., Sommer N.G., Schwarze U.Y., Marek R.L., Brcic I., Foessl I., Leithner A., Seibert F.J., Herber V., Holweg P.L. (2024). Mg-Zn-Ca alloy (ZX00) screws are resorbed at a mean of 2.5 years after medial malleolar fracture fixation: follow-Up of a first-in-humans application and insights from a sheep model. Clin. Orthop..

