# Supplementary material for: A high-toughness, rare-earth-free magnesium alloy with excellent biocompatibility profile: Integrated in vitro and in vivo characterization
Source: Mater Today Bio. 2026 May 13;38:103225. doi: 10.1016/j.mtbio.2026.103225 (PMC13214538; doi:10.1016/j.mtbio.2026.103225)
Supplement: Multimedia component 1 [file mmc1.docx]

# **Supplementary figures**


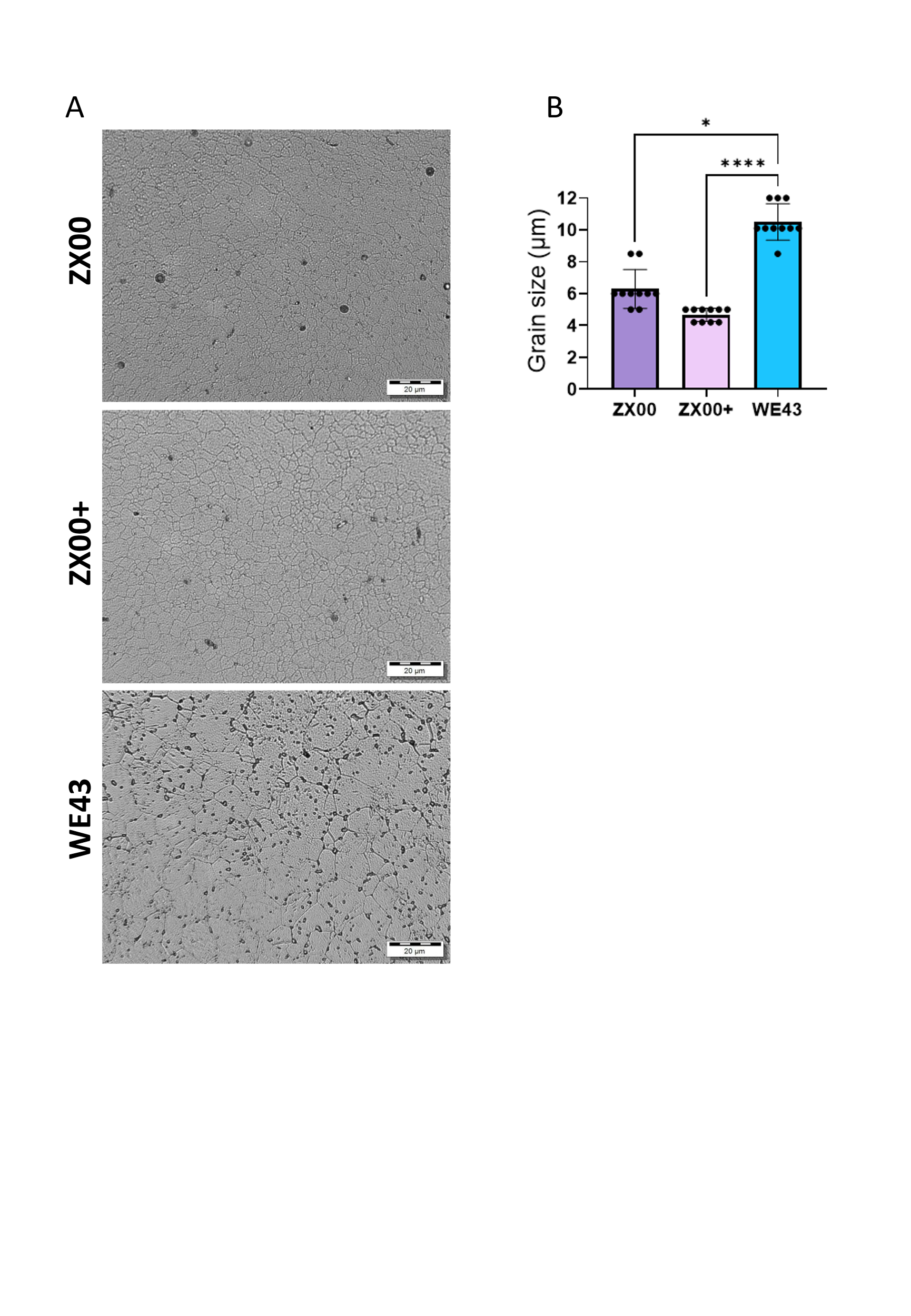


**Supplementary Figure 1**: Baseline grain size measurements. (A) Representative microscopical images of ZX00, ZX00+, and WE43 after polishing and etching. (B) Grain size measurement results. Data are shown as individual measurements and mean ± SD. **p* < 0.05, *****p* < 0.0001.


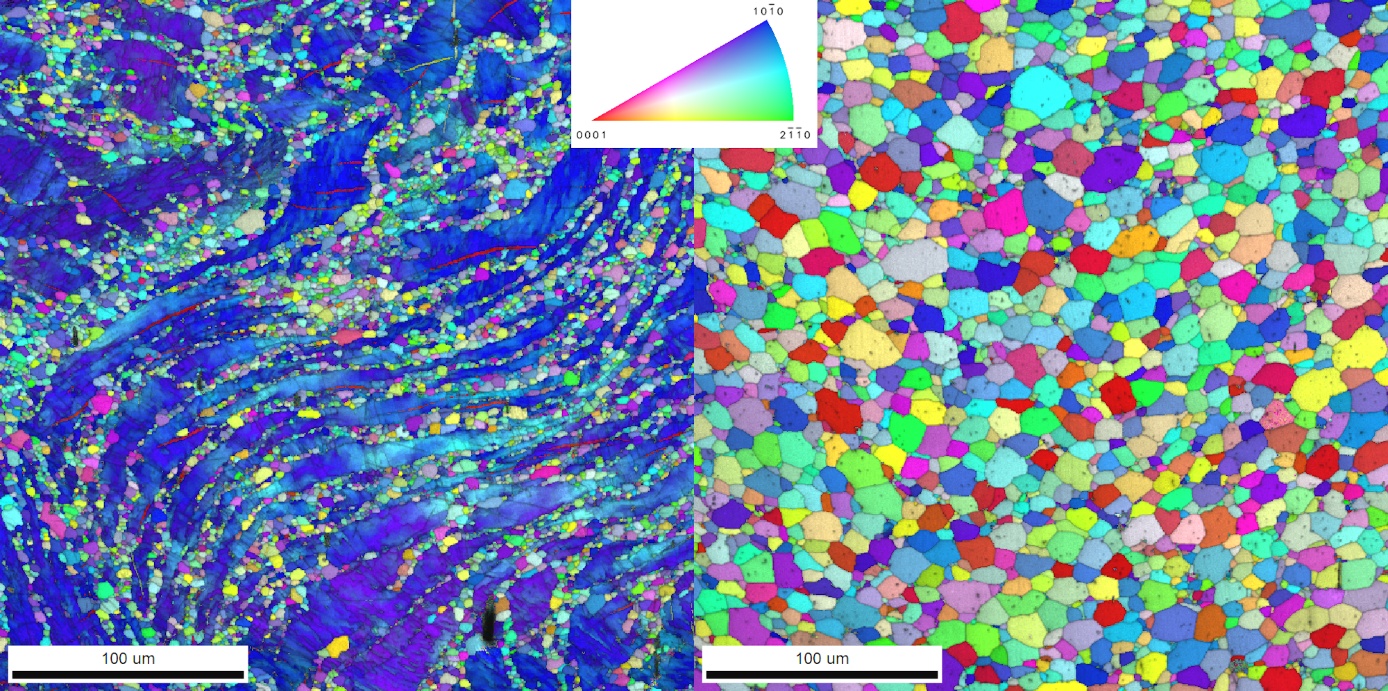


**Supplementary Figure 2**: EBSD investigations of grain orientations: Overlay of the inverse pole figure (IPF) map with respect to the sample normal direction and the image quality map. Left: ZX00; Right: WE43


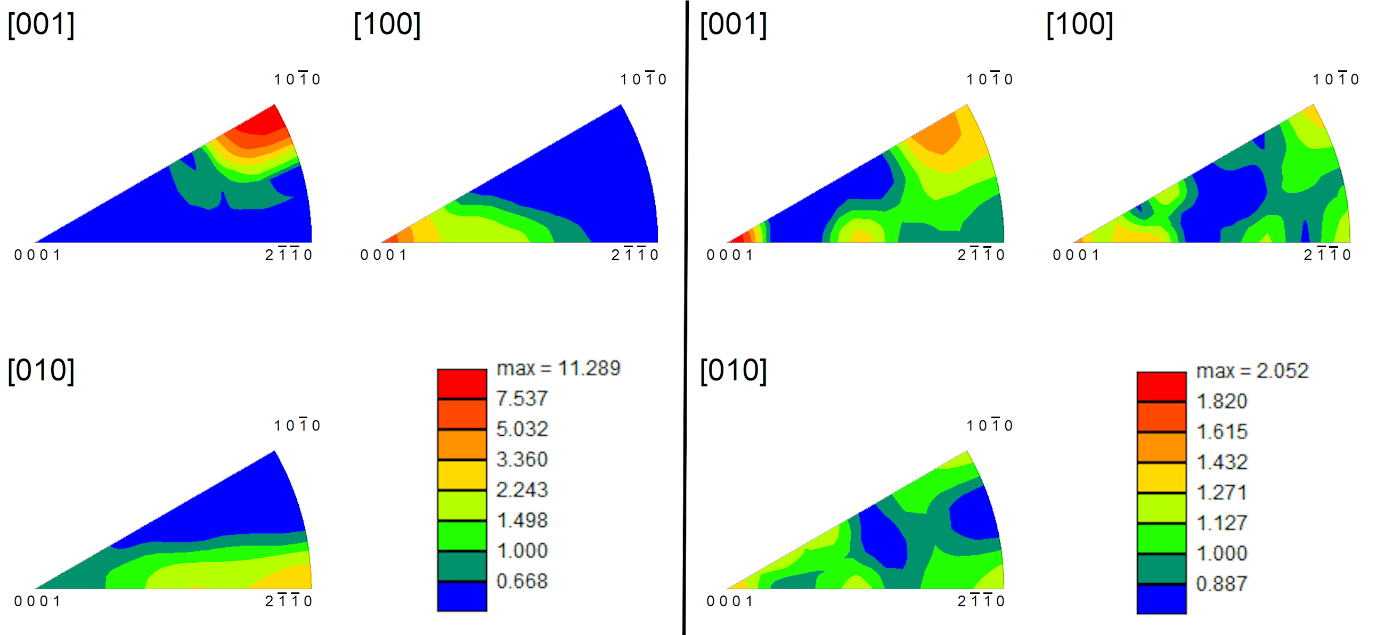


**Supplementary Figure 3:** Analyses of EBSD measurements: Inverse pole figures. Left: ZX00; Right: WE43.


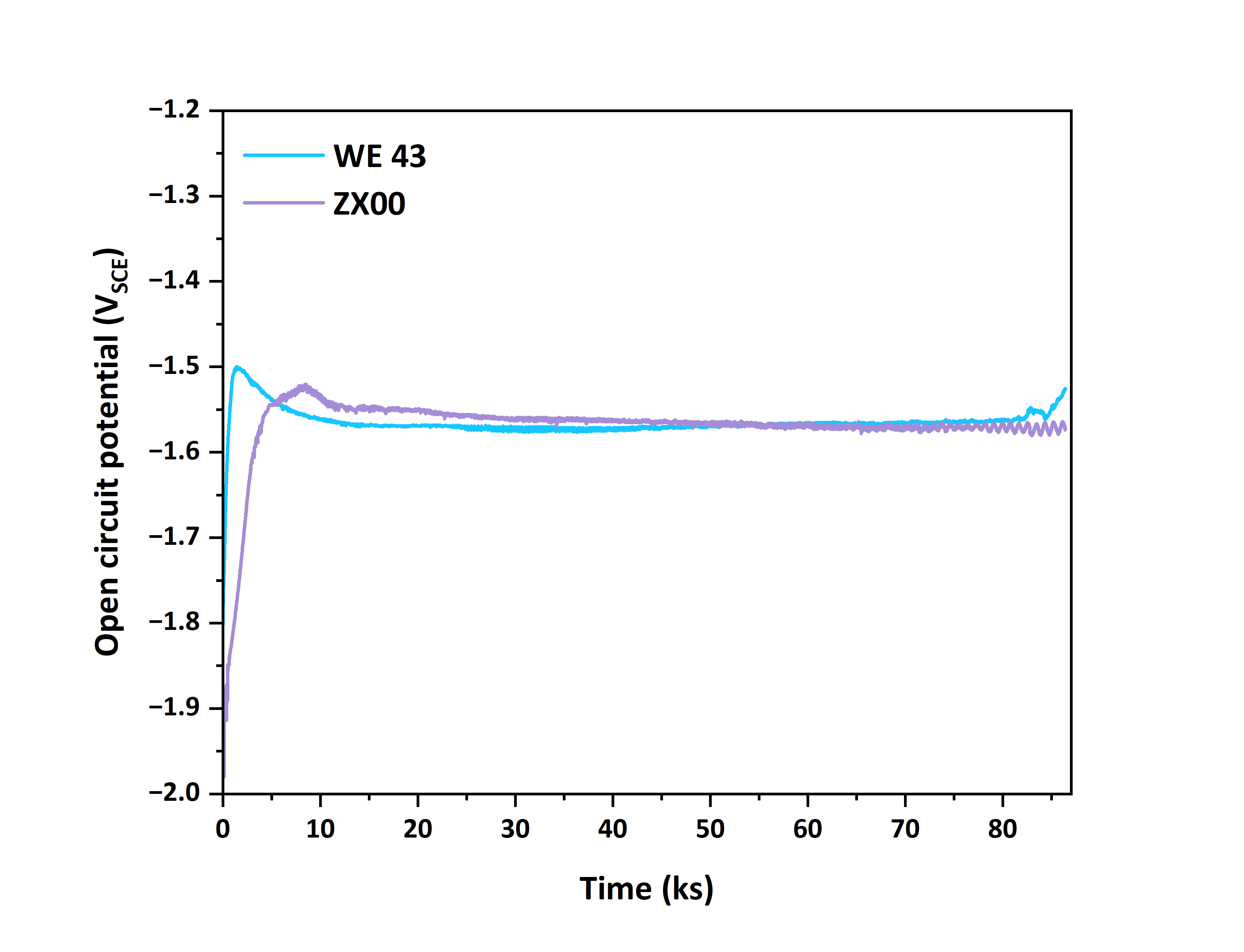


**Supplementary Figure 4**: The open circuit potential of WE43 and ZX00 alloys recorded in SBF solution at 37 °C over the immersion period of 24 hours.


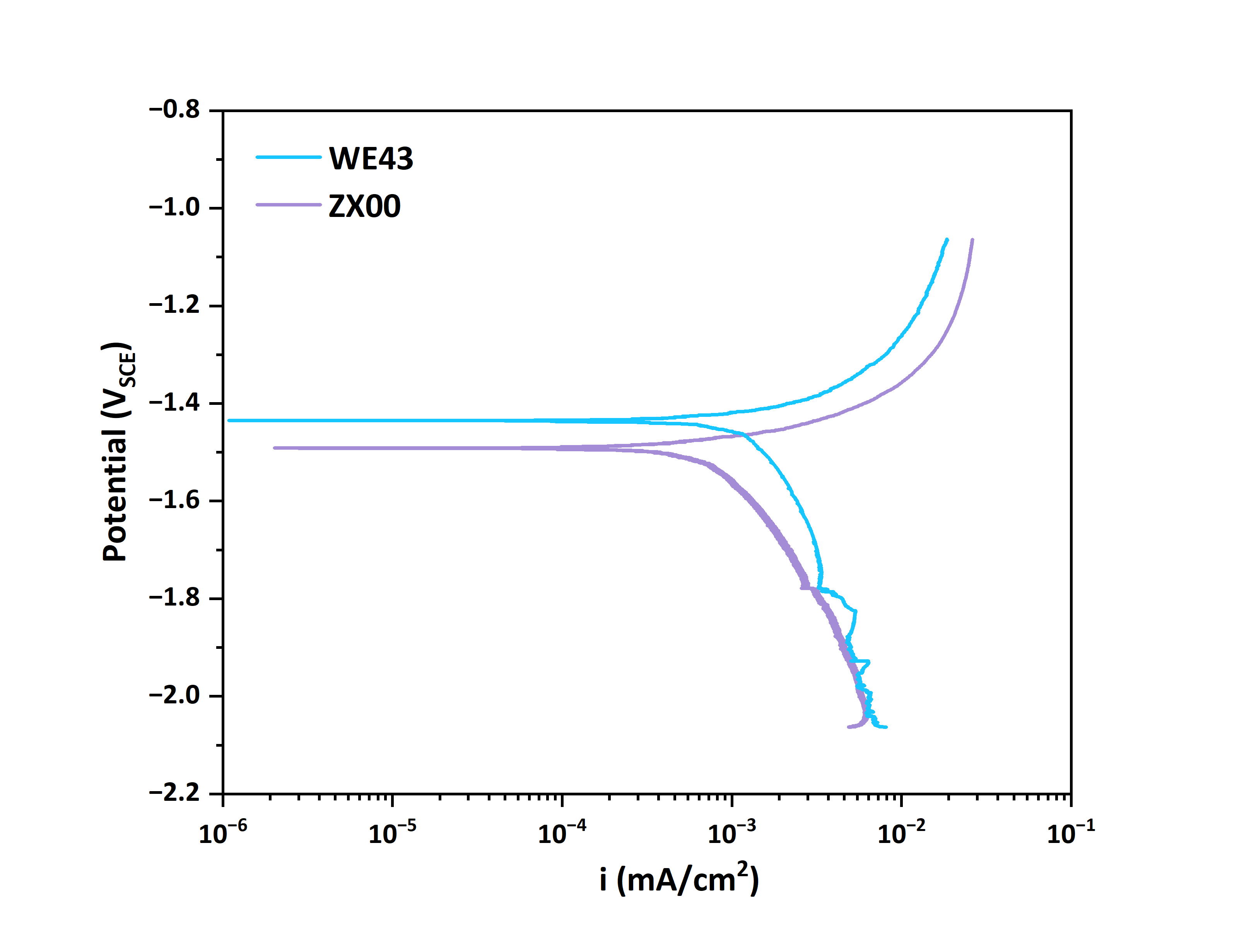


**Supplementary Figure 5**: Representative potentiodynamic polarization curves of WE43 and ZX00 measured in the range from -500 mV to +500 mV respective to OCP with scan rate of 0.05 mV/s after 24 h immersion in SBF solution at 37 °C.


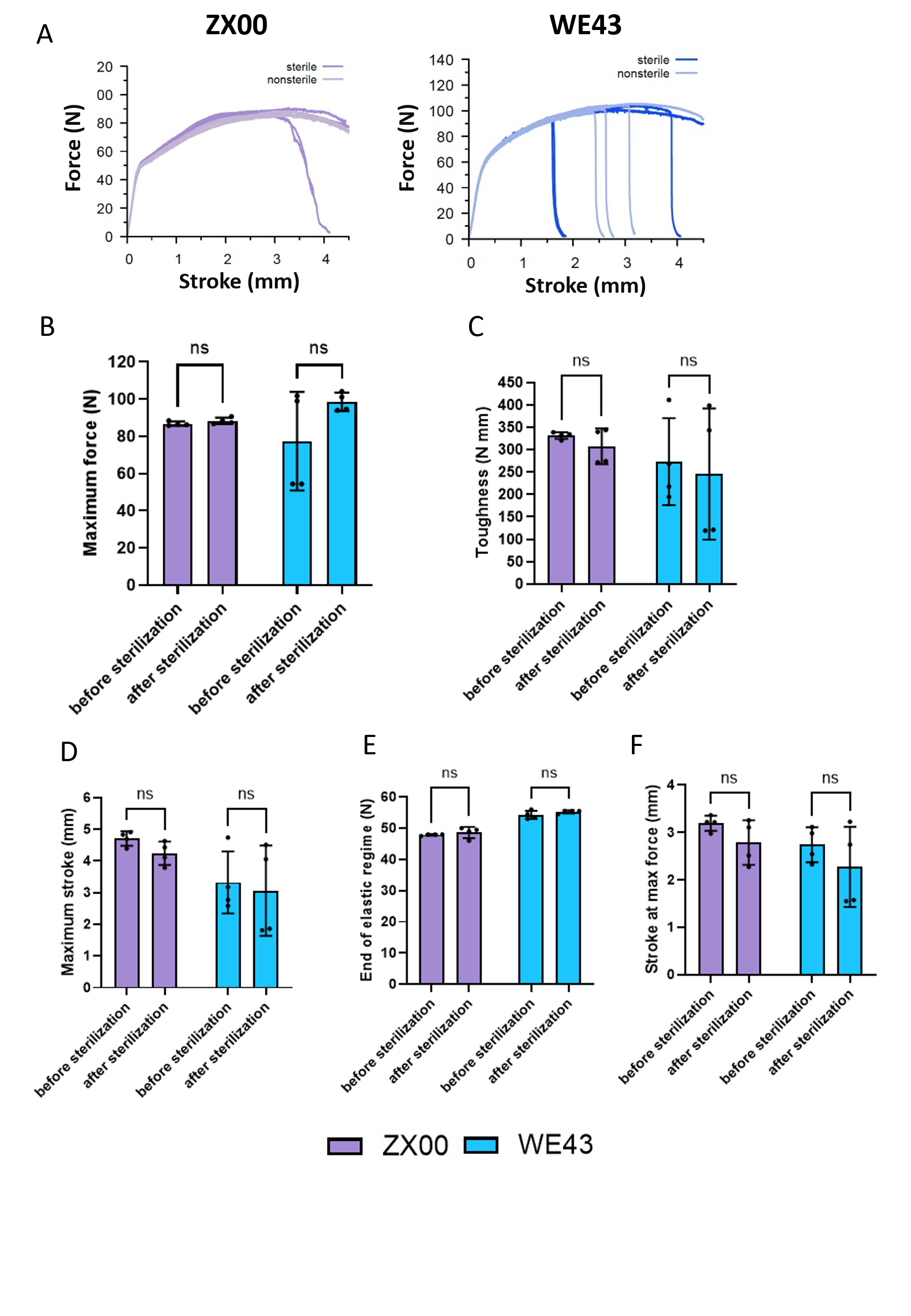


**Supplementary Figure 6**: Influence of sterilization on mechanical properties. (A) Force–stroke plots from three-point bending tests of ZX00 and WE43 rods. (B) Maximum force, (C) toughness, (D) maximum stroke, (E) end of the elastic regime, and (F) stroke at maximum force. Individual data points, mean values, and standard deviations are shown. ns = not significant.


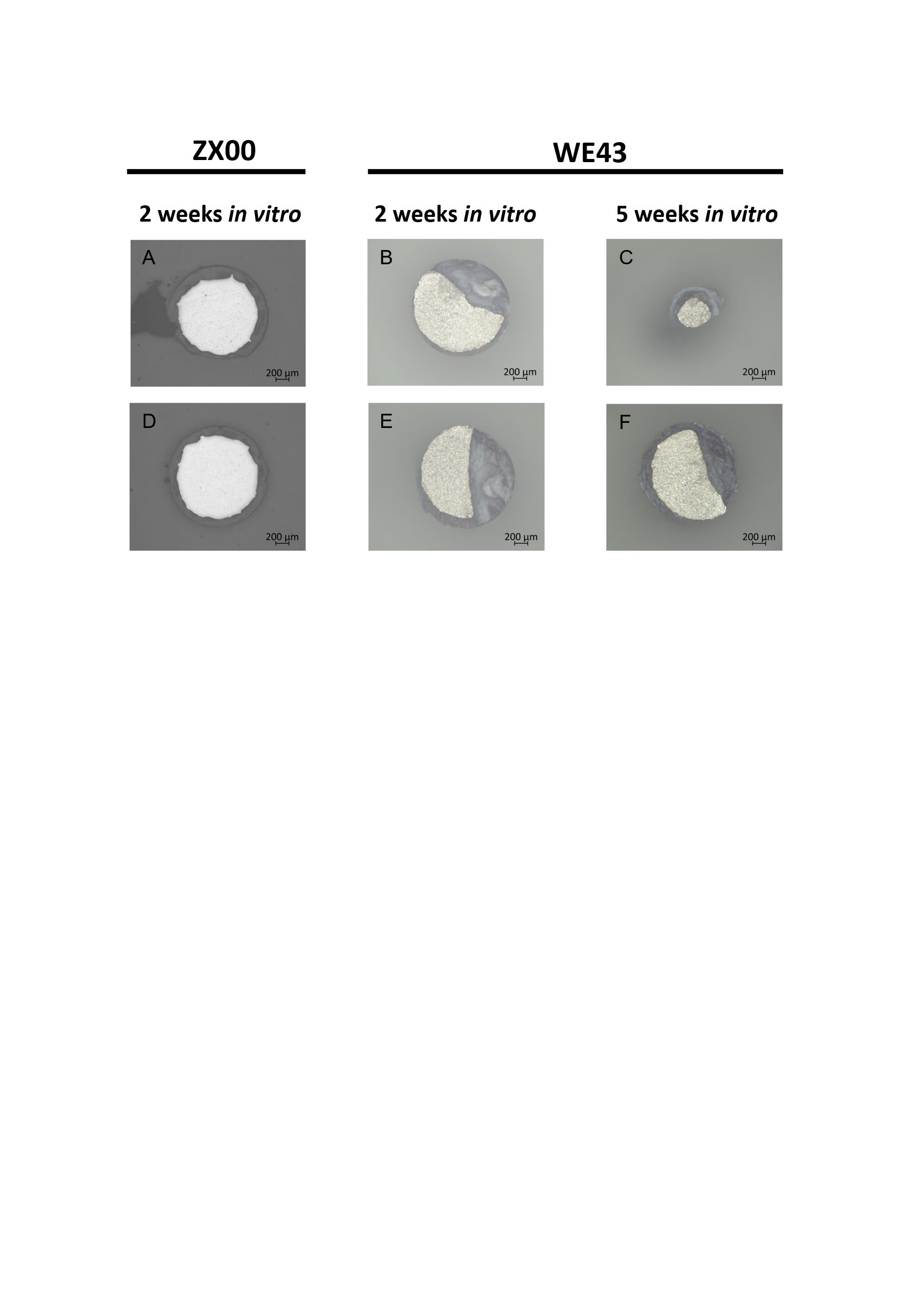


**Supplementary Figure 7**: Cross-sections and fracture surface analyses by light microscopy. (A, D) Cut and polished cross-sections of ZX00 pins after 2 weeks degradation in SBF. (B, E) Fracture surfaces of WE43 pins after 2 weeks degradation in SBF. (C, F) Fracture surfaces of WE43 after 5 weeks degradation in SBF.


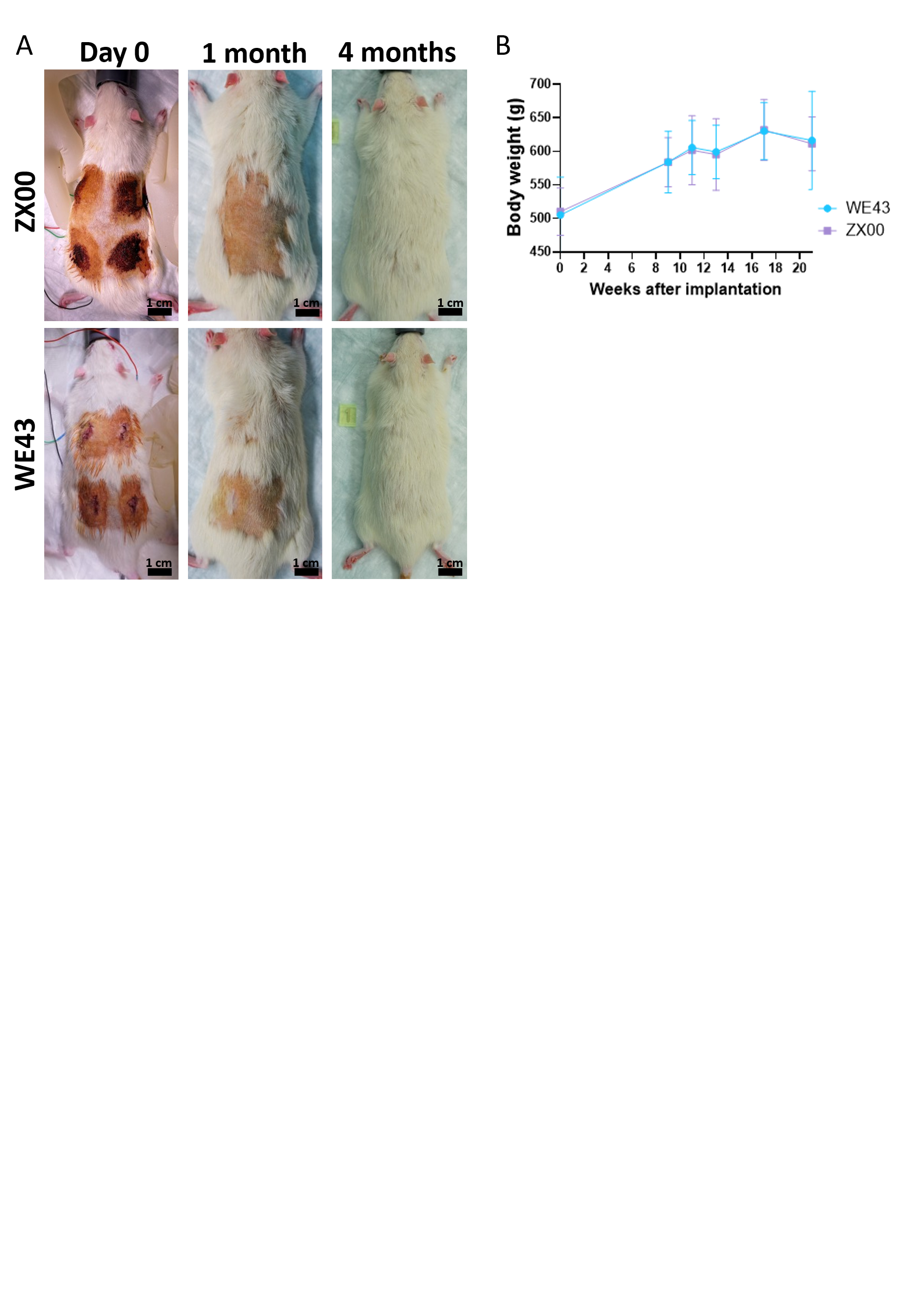


**Supplementary Figure 8**: Postoperative recovery of ZX00- and WE43-implanted rats. (A) Representative images on the day of implantation and at 1 and 4 months post-implantation. (B) Body weight changes from the day of implantation until explantation at 5 months. Mean values and standard deviations are shown.

**Supplementary tables**

**Supplementary Table 1:** Corrosion potential (Ecorr), corrosion current density (icorr), and cathodic Tafel slope values obtained for alloys WE43 and ZX00 after immersion in SBF solution for 24 h. Mean values and standard deviations.

| Material | Corrosion potential (V_SCE_) | Corrosion current density  (μA/cm^2^) | Cathodic Tafel slope  (mV/dec) |
| --- | --- | --- | --- |
| WE43 | -1.400 ± 0.05 | 2.73 ± 0.75 | -321.5 ± 42.9 |
| ZX00 | -1.520 ± 0.04 | 2.64 ± 0.23 | -323.5 ± 42.9 |

**Supplementary Table 2:** PCR primer specifications for human macrophage and rat skin tissue specimens.

| Species | Gene | Forward (5ʹ→3ʹ) | Reverse (5ʹ→3ʹ) |
| --- | --- | --- | --- |
| Human | ***CCR7*** | GCT GGT CTG AGC ATT TGA GT | CCA GAA ACT GTT CCC ACC TG |
| Human | ***CD68*** | CCT GGG TTG CTA ACC ATC TCC | CCA GTG CTC TCT GTA ACC GT |
| Human | ***CD80*** | CGG GAA ACA CTG CTA GTA CC | AAA GCC TTT AGA GCC AGC CC |
| Human | ***CD86*** | GGA ACC AAG CAA GAG CAC TG | ATG CCA GGA CTG ACG GTA GA |
| Human | ***CD163*** | CTC CTT ACT CTC CTG ATG CCA A | TGA AGA CTC TGG ATC TGC TGG T |
| Human | ***CD206*** | CTT AGT TCC GCC CTC CTG TC | TGC CTC AGA GCA GAC TTA CC |
| Human | ***GAPDH*** | GGA AGG AAA TGA ATG GGC AG | CCC AAT ACG ACC AAA TCA GAG |
| Human | ***IL-1α*** | CAG TGG TCT CAT GGT TGT CA | CTT GAG CCA GTA ATT GGT CCG |
| Human | ***IL-1β*** | CTT CGA GGC ACA AGG CAC AA | TTC ACT GGC GAG CTC AGG TA |
| Human | ***IL-10*** | CTC AGC ACT GCT CTG TTG CCT | AGC TGT TCT CAG ACT GGG TGC |
| Human | ***TNFα*** | TCC CCA GGG ACC TCT CTC TA | GGG TTT GCT ACA ACA TGG GC |
| Rat | ***CCR7*** | TTT GAG CCT TTG TGA ACG GG | AAG ACA TGT GTA ACC TGC ACCA |
| Rat | ***CD68*** | CTC ATT CCC TTA CGG ACA GC | ATT CTG CGC TGA GAA TGT CC |
| Rat | ***CD80*** | TTC GGG CCT GAA GAA GCA TTA | GCC AGG GTA GTG CTA GGT TT |
| Rat | ***CD86*** | AAG ACA TGT GTA ACC TGC ACC A | TTG AGC CTT TGT GAA CGG GC |
| Rat | ***CD163*** | GAC AGA CCC AAC GGC TTA CA | TCT TAA ATG CCA ACC CGA GGA |
| Rat | ***IL-1α*** | AGG GAG TCA ACT CAT TGG CG | AGA CAG ATG GTC AAT GGC AGA |
| Rat | ***IL-1β*** | TGG CAA CTG TCC CTG AAC TC | CCC AAG TCA AGG GCT TGG AA |
| Rat | ***IL-10*** | GCA GTG GAG CAG GTG AAG AA | GTA GAT GCC GGG TGG TTC AA |
| Rat | ***MRC1*** | TGA TTC CGG TCG CTG TTC AA | GAA CGG AGA TGG CGC TTA GA |
| Rat | ***TNFα*** | GTG ATC GGT CCC AAC AAG GA | CTT GGT GGT TTG CTA CGA CG |

**Supplementary Table 3:** Antibodies used for macrophage immunofluorescence staining.

| **Primary Antibody** | **Source** | **Dilution** | **Secondary Antibody** | **Supplier** |
| --- | --- | --- | --- | --- |
| **CD68**  Cat. No.: ab201340 | Mouse | 1:200 | Goat Anti-Mouse IgG H&L (Alexa Fluor® 647), ab150115 | Abcam, Cambridge, UK |
| **CCR7**  Cat. No.: ab253187 | Rabbit | 1:200 | Goat Anti-Rabbit IgG H&L (Alexa Fluor® 488), ab150077 | Abcam, Cambridge, UK |
| **CD163**  Cat. No.: ab87099 | Rabbit | 1:200 | Goat Anti-Rabbit IgG H&L (Alexa Fluor® 488), ab150077 | Abcam, Cambridge, UK |

**Supplementary Table 4:** Antibodies used for immunohistochemical staining of rat skin tissue.

| **Primary Antibody** | **Source [Clone]** | **Pre-treatment** | **Dilution** | **Secondary Antibody** | **Supplier** |
| --- | --- | --- | --- | --- | --- |
| **CD3**  Cat. No.: A0452 | Rabbit [poly] | 30 min Tris-EDTA, pH 9.0 (steamer) | 1:1000 | BrightVision PolyHRP-anti-rabbit (Immunologic, Duiven, The Netherlands) | Agilent Technologies, Santa Clara, CA, USA |
| **CD20**  Cat. No.: ab27093 | Rabbit [poly] | None | 1:50 | BrightVision PolyHRP-anti-rabbit (Immunologic, Duiven, The Netherlands) | Abcam, Cambridge, UK |
| **CD68**  Cat. No.: MCA341R | Mouse [ED1] | 1 mg/mL Protease, 20 min, room temperature | 1:500 | BrightVision PolyHRP-anti-mouse (Immunologic, Duiven, The Netherlands) | Bio-Rad, Vienna, Austria |
| **CD163**  Cat. No.: LS-B5967 | Mouse [ED2] | 1 mg/mL Protease, 20 min, room temperature | 1:100 | BrightVision PolyHRP-anti-mouse (Immunologic, Duiven, The Netherlands) | LSBio, Lynnwood, WA, USA |
| **Calprotectin**  Cat. No.: MS-148 | Mouse [Mac387] | 1 mg/mL Protease, 20 min, room temperature | 1:500 | BrightVision PolyHRP-anti-mouse (Immunologic, Duiven, The Netherlands) | Thermo Fisher Scientific, Waltham, MA, USA |

**Supplementary Table 5:** Incidence of microscopic findings with grades.

| **Implantation group** | **WE43** | **ZX00** |
| --- | --- | --- |
| number of animals | 4 | 4 |
|  |  |  |
| **Brain** |  |  |
| number examined | 4 | 4 |
|  |  |  |
| **Heart** |  |  |
| number examined | 4 | 4 |
|  |  |  |
| **Lungs** |  |  |
| number examined | 4 | 4 |
|  |  |  |
| **Lymphoid hyperplasia (BALT)** |  |  |
| (minimal) | 2 | 2 |
| (slight) | 2 | 1 |
| Total | 4 | 3 |
|  |  |  |
| **Liver** |  |  |
| number examined | 4 | 4 |
|  |  |  |
| **Inflammatory infiltrate, lymphoid** |  |  |
| (minimal) | 3 | 2 |
| (slight) | 0 | 2 |
| Total | 3 | 4 |
|  |  |  |
| **Kidneys** |  |  |
| number examined | 4 | 4 |
| **Cystic tubules** |  |  |
| (minimal) | 0 | 1 |
| **Hyaline cast(s)** |  |  |
| (minimal) | 0 | 1 |
| **Inflammatory infiltrate, lymphoid** |  |  |
| (minimal) | 3 | 2 |
| **Tubular basophilia, corticomedullary** |  |  |
| (minimal) | 1 | 0 |
| **Spleen** |  |  |
| number examined | 4 | 4 |
| **Hemopoietic foci, primarily erythroid** |  |  |
| (minimal) | 1 | 1 |
| **Hemosiderin pigment** |  |  |
| (minimal) | 0 | 2 |
| (slight) | 1 | 0 |
| (moderate) | 3 | 2 |
| Total | 4 | 4 |
